# Individual determinants of research utilization by nurses: a systematic review update

**DOI:** 10.1186/1748-5908-6-1

**Published:** 2011-01-05

**Authors:** Janet E Squires, Carole A Estabrooks, Petter Gustavsson, Lars Wallin

**Affiliations:** 1 Clinical Epidemiology Program, Ottawa Hospital Research Institute, Ottawa, Ontario, Canada; 2 Faculty of Nursing, University of Alberta, Edmonton, Alberta, Canada; 3 Division of Psychology, Department of Clinical Neuroscience, Karolinska Institutet, Stockholm, Sweden; 4 Division of Nursing, Department of Neurobiology, Care Sciences and Society, Karolinska Institutet; and Clinical Research Utilization (CRU), Karolinska University Hospital, Stockholm, Sweden

## Abstract

**Background:**

Interventions that have a better than random chance of increasing nurses' use of research are important to the delivery of quality patient care. However, few reports exist of successful research utilization in nursing interventions. Systematic identification and evaluation of individual characteristics associated with and predicting research utilization may inform the development of research utilization interventions.

**Objective:**

To update the evidence published in a previous systematic review on individual characteristics influencing research utilization by nurses.

**Methods:**

As part of a larger systematic review on research utilization instruments, 12 online bibliographic databases were searched. Hand searching of specialized journals and an ancestry search was also conducted. Randomized controlled trials, clinical trials, and observational study designs examining the association between individual characteristics and nurses' use of research were eligible for inclusion. Studies were limited to those published in the English, Danish, Swedish, and Norwegian languages. A vote counting approach to data synthesis was taken.

**Results:**

A total of 42,770 titles were identified, of which 501 were retrieved. Of these 501 articles, 45 satisfied our inclusion criteria. Articles assessed research utilization in general (n = 39) or kinds of research utilization (n = 6) using self-report survey measures. Individual nurse characteristics were classified according to six categories: beliefs and attitudes, involvement in research activities, information seeking, education, professional characteristics, and socio-demographic/socio-economic characteristics. A seventh category, critical thinking, emerged in studies examining kinds of research utilization. Positive relationships, at statistically significant levels, for general research utilization were found in four categories: beliefs and attitudes, information seeking, education, and professional characteristics. The only characteristic assessed in a sufficient number of studies and with consistent findings for the kinds of research utilization was attitude towards research; this characteristic had a positive association with instrumental and overall research utilization.

**Conclusions:**

This review reinforced conclusions in the previous review with respect to positive relationships between general research utilization and: beliefs and attitudes, and current role. Furthermore, attending conferences/in-services, having a graduate degree in nursing, working in a specialty area, and job satisfaction were also identified as individual characteristics important to research utilization. While these findings hold promise as potential targets of future research utilization interventions, there were methodological problems inherent in many of the studies that necessitate their findings be replicated in further research using more robust study designs and multivariate assessment methods.

## Background

In this paper, we update the evidence published in a previous systematic review on individual characteristics that influence nurses' use of research evidence in clinical practice. Research utilization refers to 'that process by which specific research-based knowledge (science) is implemented in practice' [1]. In recent years, research utilization by nurses has received increased attention in the literature and has been conceptualized and measured in terms of four kinds or types of research use: instrumental, conceptual, persuasive (or symbolic), and overall [1-3]. Instrumental research utilization refers to the concrete application of research findings in clinical practice. Conceptual research utilization refers to the cognitive use of research where the research may be used to change one's thinking about a specific practice, but may or may not result in a change in action. Persuasive or symbolic research utilization is the use of research as a persuasive or political tool to legitimate a position or influence the practice of others. Overall research utilization is an omnibus construct and refers to the use of any kind of research in any way [1,4].

Research utilization scholars continuously express concern about whether nurses use the best available scientific (*i.e*., research) evidence to guide their clinical practice [4-7]. This disparity between the availability of research evidence and its use in practice is often referred to as the 'research-practice gap.' The nature of this gap has been the subject of debate in the nursing literature. Larsen *et al*. [8], for example, have argued that there is no theory-practice gap; that the knowledge forms at issue in theory-practice gap discourse are radically different in kind. This stands in contrast to the views of other well-respected theorists (*e.g*., Allmark [9] and Fealy [10]) who articulate the nature of the gap, its origins, and in some cases, solutions to it. While, several examples of the research-practice gap have been highlighted in the nursing literature, most of the evidence is anecdotal due to difficulties surrounding attempts to measure whether or not nursing practice is research-based [11]. It remains generally accepted however that a research-practice gap exists.

Despite increased knowledge of the benefits of adopting a research-based approach to providing nursing care and of increased availability of research findings for nurses, the use of research findings in nursing practice remains, at best, slow and haphazard [12-14]. As a result, patients frequently do not receive best (or even optimal) nursing care. In response, there is an accelerated research agenda calling for the implementation of interventions to increase research use by nurses. However, relatively few reports of research utilization interventions in nursing exist and more importantly, where they do exist, positive findings are generally not reported [15]. One review examining interventions to increase research utilization by nurses has been published. Thompson *et al*. [16] concluded findings on the effectiveness of interventions to increase research use in nursing are equivocal and at best, a combination of educational interventions and local opinion leaders or multidisciplinary teamwork may be effective. One reason for this relative lack of knowledge on successful research utilization interventions in nursing, we argue, is the lack of systematic identification and evaluation of factors (individual, contextual, and organizational) associated with research utilization.

In a previous systematic review of individual characteristics related to research utilization by nurses, Estabrooks *et al*. [17] identified 95 characteristics that they grouped into six core categories: beliefs and attitudes, involvement in research activities, information seeking, education, professional characteristics, and other socio-economic factors. The six categories were not predetermined but emerged from the data extraction. By using a vote-counting approach to synthesis, Estabrooks *et al*. [17] concluded the most frequently studied individual characteristic and the only one with a consistently positive effect was 'attitude towards research', which is part of the larger category 'beliefs and attitudes.' Findings for other individual characteristics were highly equivocal and were characterized by serious study design and methodological flaws. In this paper, we update the evidence on individual characteristics of research utilization by searching additional electronic databases and by adding the results of studies published between 2001 and 2008 to the evidence reported in the previous review. We also expand on the previous review by reporting on the magnitude of effect between individual nurse characteristics and research utilization and by searching for and examining literature on kinds of research utilization (*i.e*., instrumental, conceptual, persuasive, overall) with respect to individual characteristics important to research utilization in nursing.

## Methods

### Selection criteria for studies

#### Types of study

Randomized controlled trials, clinical trials, and observational (*i.e*., quasi-experimental, cohort, case-control, cross-sectional) designs that examined the association between individual characteristics and nurses' use of research in practice were eligible for inclusion. Case reports and editorials were excluded. Studies were further limited to those published in the English, Danish, Swedish, and Norwegian languages. There were no restrictions on the basis of country of origin, when the study was undertaken, or publication status.

#### Type of participant, characteristic, and outcome

We considered studies that examined relationships between individual characteristics and nurses' use of research. A nurse was defined as a professional who provides care in a clinical setting; this definition includes registered nurses, licensed practical nurses, nurse leaders, and clinical nurse educators. All individual characteristics, modifiable and non-modifiable, were eligible for inclusion. The outcome of interest was research utilization. We defined research utilization as the use of research-based information -- that is, information that is empirically derived. This information could be reported in a primary research article, review/synthesis report, or protocol. If the study involved the use of a protocol, the authors were required to make the research-basis for the protocol apparent in the report. We excluded articles that reported on: the adherence to clinical practice guidelines, rationale being that clinical practice guidelines can be based on non-research evidence (*e.g*., expert opinion), and the use of one specific-research-based practice if the purpose was not to examine nurses' use of research in practice generally. We did include nurses' use of protocols where the research-base of the protocol was made explicit in the research report. We also required that the relationship between the individual characteristic(s) and research utilization be expressed quantitatively (and tested statistically).

### Search strategy for identification of studies

This review was conducted as part of a larger review on research utilization instruments [18]. The objectives of the larger review are: to identify instruments used to measure research utilization by healthcare providers, healthcare decision makers, and in healthcare organizations; and to assess the psychometric properties of these instruments. Research utilization instruments refer to self-report measures that assess healthcare providers' and decision makers' use of research-based knowledge in their daily practice. We searched the following 12 online bibliographic databases: Cochrane Database of Systematic Reviews (CDSR), Health and Psychosocial Instruments (HAPI), MEDLINE, CINAHL, EMBASE, Web of Science, SCOPUS, OCLC Papers First, OCLC WorldCat, Sociological Abstracts, Proquest Dissertation Abstracts, and Proquest ABI Inform. Key words and medical subject headings related to research utilization were identified prior to initiating the search. Additional File 1 displays a summary of the search strategy used in the larger review. We also hand searched the journals *Implementation Science *(a specialized journal in the research utilization field) and *Nursing Research *as well as the bibliographies of articles identified for inclusion in the review.

### Study identification and quality assessment

One investigator (JES) and a research assistant screened the titles and abstracts of the articles identified by the search strategy. Articles that potentially met our inclusion criteria, or where there was insufficient information to make a decision regarding inclusion, were retrieved and assessed for relevance by one investigator (JES) and a research assistant. Disagreements throughout the selection process were resolved by consensus. To assess methodological quality of the final set of articles, we adapted two previously used tools: Estabrooks' Quality Assessment and Validity Tool for Cross-Sectional Studies, and the Quality Assessment Tool for Quantitative Studies. Each article had a quality appraisal performed by two reviewers. Articles were classified as weak, moderate-weak, moderate-strong, or strong using a system developed based on work by De Vet *et al*. [19] that has been used in other published systematic reviews [17,20,21]. All discrepancies in quality assessment were resolved through consensus.

Estabrooks' Quality Assessment and Validity Tool was developed based on the Cochrane Collaboration guidelines (in existence in 2001) and medical literature [22,23]. The tool contains a maximum of 16 total points covering three core domains: sample, measurement, and statistical analysis (Additional File 2). In order to derive a final score for each of the included articles (cross-sectional design), the total number of points obtained was divided by the total number of possible points, allowing for a score between 0 and 1 for each article. The articles were then classified as weak (<0.50), moderate-weak (0.51 to 0.65), moderate-strong (0.66 to 0.79), or strong (0.80 to 1.00).

The Quality Assessment Tool for Quantitative Studies Tool, developed for the Canadian Effective Public Health Practice Project, has been judged suitable to be used in systematic reviews of interventions [24,25]. The tool contains a maximum of 18 total points covering six content areas: selection bias (is the study sample representative of the target population), allocation bias (extent that assessments of exposure and outcome are likely to be independent), confounders (were important confounders reported and appropriately managed), blinding (were the outcome assessor(s) blinded to the intervention or exposure status of participants), data collection methods (reliability and validity of data collection methods and instruments), and withdrawals and dropouts (percentage of participants completing the study) (Additional File 3). Each article is scored as weak, moderate, strong, or not applicable in each of these six areas according to preset criteria that accompany the tool. The tool developers do not provide a means for calculating an overall quality score. However, in order to compare the quality scores for each included article that used an intervention design (assessed with this tool) to the included articles that used cross-sectional designs (assessed with Estabrooks' Quality Assessment and Validity Tool), we derived an overall quality score for each article. To derive this score, we assigned values of 1, 2, and 3 to the categorizations of weak, moderate, and strong in each content area respectively. A final quality score for each article was then obtained by dividing the summative score obtained by the number of applicable content areas (*i.e*., by 6 - the number of points not applicable for the article). The articles were then classified as weak (1.0 to 2.0), moderate-weak (2.1 to 2.34), moderate-strong (2.35 to 2.66), or strong (2.67 to 3.0).

### Data extraction and analysis

One reviewer (JES) extracted data from all included articles. Extracted data was double checked by a research assistant for accuracy. Data were extracted on study design, objectives, sample and subject characteristics, theoretical framework, instruments used, reliability, validity, and key findings with respect to relationships between individual characteristics and nurses' research utilization (Tables 1 and 2 and Additional File 4). All discrepancies in data extraction were resolved through consensus.

**Table 1:** Summary of findings for studies reporting research utilization in general (n = 39 articles)

Individual Determinant	First Author	Significance*	Direction and Magnitude	Comment
**1. BELIEFS AND ATTITUDES**				

Perceived support for research	Butler [71]	NS		

Attitude toward research	Champion [50]	S	+ (r = .55)	
	Estabrooks [31]	S	+ LISREL	Chi square = 55.91 p = .263 for model with attitude, belief suspension and in-services
	Hatcher [52]	S	+ (r = .65 - .82)	
	Lacey [54]	S	+ (r = .674)	
	Prin [56]	S	+ (r = .58)	
	Tranmer [57]	S	+ (β = .64)	
	Varcoe [61]	S	+ (r = .41)	S for general research use (RUQ); NS for specific practices
	Wells [72]	S	+ (β = 1.62)	

Expectation of self to use research	Varcoe [61]	S	+ (r = .51)	With general use of research (not specific findings)

Expressed interest in research	Varcoe [61]	S	+ (r = .50)	With general use of research (not specific findings)

Problem solving ability	Estabrooks [31]	NS		

Cosmopoliteness	Estabrooks [31]	NS		

	Estabrooks [31]	NS		
Autonomy	Forbes [62]	S	+ (r = 0.08)	
	McCloskey [33]	S	*+ (*β *= *0.135)	

Dogmatism	Estabrooks [31]	NS		

Activism	Estabrooks [31]	NS		

Belief suspension	Estabrooks [31]	S	+ (LISREL)	Chi square = 55.91 p = .263 for model with attitude, in-services, belief suspension

Theoretical orientation	Estabrooks [31]	NS		

Trust	Estabrooks [31]	NS		

Confidence	Wells [72]	NS		Confidence in research related activities (*e.g*., reading research, discussing research)

Career commitment	Stiefel [60]	S	+ (R^2 ^= 0.13)	MANOVA

Perception of nurse as a RU barrier	Bostrom [51]	S	+ (*t *= 2.512)	Research user reports less individual barriers

Awareness (overall) of practice	Squires [14]	S	+ (β = 2.52)	For 'user of research'

Awareness of practice by regular use	Squires [14]	S	+ (β = 3.49)	For 'user of research'

Research awareness	Wells [72]	NS		

Persuaded (believe in) of the practice	Squires [14]	S	+ (β = 2.11)	For 'user of research'

**2. INVOLVEMENT IN RESEARCH ACTIVITIES**				

Current data collection for others	Butler [71]	S	+ (OR = 4.04)	

Participation in research-related activities	Berggren [46]	NS		
	McCleary [29]	S	+	Test statistic not given

Participation in research as subject	Hatcher [52]	NS		
Past use of research	Butler [71]	S	+ OR = 20.0	

Job related research activities	Rutledge [49]	S	+ (r = .0673 to .1272)	S for 3 of 8 practices

Participation in research study	Brett [44]	NS		

	Nash [55]	NS		

Education for research participation	Logsdon [77]	S	+ (r = .32)	

Research participation	Tsai [74]	S	+ (r = .3268)	

Involvement in research projects	Tranmer [57]	NS		

Research experience	Varcoe [61]	S	+ (r = .37)	With general use of research (not specific findings)

Participation in quality management	McCleary [29]	S	+	Test statistic not given

Participation in quality improvement	Wallin [58]	S	+ (*X*^2 ^= 11.1)	

Completion of the research study	Tsai [75]	NS		

**3. INFORMATION SEEKING**				

Nursing texts as information	Barta [45]	NS		

Nursing journals as information \	Barta [45]	S	+ (t = -2.36)	

Education by specialty groups	Barta [45]	NS		

Personal experience as information	Squires [14]	S	+ (β = 0.55)	For 'consistent research user'

P&P manual as information	Squires [14]	NS		

In-services as a source of knowledge	Squires [14]	NS		

Attended education program	Berggren [46]	NS		

Critical reading skills	Tranmer [57]	S	+ (β = 0.19)	Pre-test & Post-test respondents combined

Use computer	Wallin [69]	S	+ (β = 0.142)	

Time per week on the internet	Wallin [69]	NS		

Internet use	Cummings [68]	NS		

Have a personal computer	Wallin [69]	NS		

Reading activities Read journals				
	Berggren [46]	NS		

Hours reading journals	Brett [44]	S	+ (r = .163)	
	Coyle [47]	NS		
	Michel [48]	NS		

Number of journals read	Rodgers [12]	S	+ (Z = 2.98)	
	Rutledge [49]	S	+ (r = .0901)	1 of 8 practices
	Wells [72]	NS		

Reads *Heart & Lung*	Coyle [47]	S	+ (*X*^2 ^= 3.795)	
	Michel [48]	S	+ Mann Whitney U = 1422.0	

Reads *Nursing Research*	Brett [44]	S	+ (*X*^2 ^= 12.422)	
	Michel [48]	NS		

Reads *RN*	Brett [44]	S	+ *(X*^2 ^= 8.925)	

Attendance at conferences/in-services	Butler [71]	NS		
	Coyle [47]	S	+ (*X*^2 ^= 5.179)	To total TIAB score
	Estabrooks [31]	S	+ (LISREL)	Chi square = 55.91 p = .263 for model with attitude, belief suspension and in-services
	Michel [48]	S	+ Mann Whitney U = 1291.5	
	Rutledge [49]	S	+ (r = .1168)	All 8 practices combined

Hours of continuing education	Brett [44]	NS		
	Coyle [47]	NS		

Number of study days attended	Rodgers [12]	S	+ (r = .095)	

Time spent studying (on duty)	Rodgers [12]	NS		

Time spent studying (off duty)	Rodgers [12]	S	+ (r = .1)	

MEDLINE usage	Prin [56]	S	+ (r = .2526)	

**4. EDUCATION**				

Increasing levels (multiple levels: diploma, bachelors, masters, PhD; post-hoc analysis not provided)	Brett [44]	NS		Diploma, Bachelors, Masters
	
	Coyle [47]	NS		
	Lacey [54]	S	+ (r = .554)	
	Logsdon [77]	S	+ (*X*^2 ^= 7.99)	Willingness to use research to change practice
	Nash [55]	NS		
	Rodgers [12]	S	+ (rho = .12)	
	
	Rutledge [49]	S	- All Practices r = -.1205, 3 of 8 practices (.0666-.1158)	Diploma/associate, bachelors, masters, doctorate Suggested in article to be spurious due to multiple tests

Type of degree	Berggren [46]	NS		Diploma, Degree
	Brown [70]	S	+ (*X*^2 ^= 36.1)	Without bachelor's vs. with bachelors vs. graduate degree.
	Bonner [59]	S	+ (H = 11.16) Kruskal wallis	Masters degree versus lower
	Butler [71]	S	+ (OR = 1.75)	Diploma, Bachelors degree (higher for degree)
	Champion [50]	NS		Graduate compared to basic education (BN)
	Erler [46]	NS		For using lit searches in practice and in policies, Diploma versus degree
	Estabrooks [31]	NS		Diploma, Degree
	Forbes [62]	NS		Diploma, Degree
	McCleary [29]	S	+ (*F *= 8.8)	Bachelors vs. community college & graduate vs. community college
	McCloskey [33.34]	S	+ (F = 11.34)	Diploma, Bachelors, Masters
	Michel [48]	S	+ (U = 2345.0)	BSN, MSN
	Ofi [73]	NS		Diploma, Degree
	Squires [14]	NS		Diploma, Degree
	Stiefel [60]	NS		Bachelors, Graduate degree
	Tranmer [57]	NS		Diploma, Degree
	Varcoe [61]	NS		Diploma, Degree
	Wallin [69]	S	+ (r = 0.229)	Diploma, Degree

Working toward a degree	Brett [44]	NS		
	Coyle [47]	NS		

Current enrolment	Brett [44]	NS		

Well prepared in education process	Logsdon [77]	S	+ (r = .32)	With willingness to change ones practice based on research

Number of degrees	Brett [44]	NS		

Courses attended	Estabrooks [31]	NS		

Completion of research class(es)	Brett [44]	NS		
	Coyle [47]	NS		
	McCleary [30]	S	+ (t = 2.9)	
	Nash [55]	NS		
	Rodgers [12]	S	+ (Mann Whitney U = 4.44)	

Completion of statistics course	Butler [71]	NS		

Completion of research design course	McCleary [29]	S	+ (*t *= 3.9)	
	McCleary [30]	S	+ (*t *= 3.5)	

Number of statistics courses taken	Wells [72]	S	+ (β = 0.48)	

Years since basic education	Brett [44]	NS		

Years since last degree	Estabrooks [31]	NS		

Taught a topic based on research	Rodgers [12]	S	+ (Mann Whitney U = 4.93)	

Having project 2000 training	Parahoo [35]	NS		

**5. PROFESSIONAL CHARACTERISTICS**				

Full or part-time status	Butler [71]	NS		
	Wallin [69]	S	+ (β = 0.228)	For work full time

Years employed as an RN	Butler [71]	NS		
	Champion [50]	NS		
	Coyle [47]	NS		
	Estabrooks [31]	NS		
	McCleary [29]	NS		
	McCloskey [34]	NS		
	Michel [48]	NS		
	Rodgers [12]	NS		
	Squires [14]	S	+ (β = 0.07)	For 'consistent research user'
	Stiefel [60]	S	+ (r = .22)	
	Tranmer [57]	NS		
	Wallin [69]	NS		

Years in post (hospital)	Tranmer [57]	NS		

Current role	Berggren [46]	NS		Staff midwife or midwifery sister
	Bonner [59]	S	+ Kruskal Wallis (H = 12.67)	Nurse unit managers and consultant report more use than staff nurses
	Butler [71]	S	+ (OR = 5.01)	Those in leadership or advanced roles report more use than staff nurses
	Connor [66]	NS		
	Hatcher [52]	S	+ (t = 5.57)	Those in leadership of advanced roles report more use as compared to staff nurses
	McCloskey [33,34]	S	+ (F = 7.901)	Management position or advanced practice nurses vs. staff nurses
	Rodgers [12]	NS		Charge nurse vs. staff nurse
	Wallin [69]	S	- (β = -0.395)	Staff nurse versus other (staff nurses use less research)
	Wells [72]	NS		Staff nurse, nurse manager

Clinical specialty	Estabrooks [31]	NS		
	Michel [48]	NS		
	Forbes [62]	S	+ ANOVA (F = 5.370	Higher RU for critical care nurses as compared to medical/surgical or obstetrical/gynecological
	Humphris [53]	S	+ *X*^2 ^(test value not reported)	Greater number of diabetic nurse specialists implement specific findings into practice as compared to the non-nurse specialist group
	Nash [55]	S	+ ANOVA *(F *= 2.35)	Area worked (highest RU mean to lowest): Education, other, hospital inpatient, outpatient clinic, office
	Parahoo [36]	S	+ (*X*^2 ^= 3.79)	Medical vs. surgical nurses
	Squires [14]	S	- (β = -0.42)	Med-surg compared to critical care unit (med-surg use less than CC)
	Stiefel [60]	S	+ (Wilk's lambda = 0.76, F = 2.23)	Critical care higher RU than medicine, surgery, oncology
	Wright [78]	NS		Analyzed groups by practice area (general hospital, psychiatric hospital, or community mental health)

Number of memberships held	Coyle [47]	NS		

Oncology nursing society status	Rutledge [49]	S	- 2 of 8 practices (-.068, -.080)	

Oncology certification	Rutledge [49]	NS		

CFRN certification	Erler [76]	S	+ (*X*^2 ^= 9.6 - use research literature); (*x*^2 ^= 11.2 - translate findings into policies and procedures)	

Job satisfaction	Coyle [47]	S	+ (r = .18)	
	Estabrooks [31]	NS		
	Berggren [46]	NS		
	Forbes [62]	S	+ (r = 0.13)	
	Wallin [69]	S	+ (β = 0.264)	

Emotional exhaustion	Cummings [68]	S	- (magnitude varied by context)	Coefficients significant but model not. High context estimated effect = -.109; partially high context estimated effect = -.191; partially low context estimated effect = -.334; low context estimated effect = -.251

Stress	Forbes [62]	S	- (r = -0.13)	Personal job stress: Juggling expectations of other professionals and of clients
	Forbes [62]	S	- (r = -0.08)	Situational job stress: Issues such as equipment, time, and staffing

Affiliation	Estabrooks [31]	NS		

Dependant care hours	Estabrooks [31]	NS		

Hours/week worked	Estabrooks [31]	NS		
	Wallin [69]	NS		

Shift usually worked	Estabrooks [31]	NS		

Shift satisfaction	Estabrooks [31]	NS		

National certification	Stiefel [60]	NS		

**6. SOCIO-DEMOGRAPHIC AND SOCIO-ECONOMIC FACTORS**				

Age	Berggren [46]	NS		
	Butler [71]	NS		
	Champion [50]	NS		
	Cummings [68]	NS		
	Estabrooks [31]	NS		
	Lacey [54]	NS		
	McCleary [29]	NS		
	Rodgers [12]	NS		
	Wallin [69]	NS		

Married or partnered/Marital status	Estabrooks [31]	NS		

Family income	Estabrooks [31]	NS		

Health/lifestyle activity	Estabrooks [31]	NS		

Gender	Estabrooks [31]	NS		

	Stiefel [60]	NS		
	Wallin [69]	NS		

*Significance: NS = not significant, S = significant at p < 0.05

**Table 2:** Summary of findings for studies reporting kinds of research utilization (n = 6 articles)

Individual Determinant	First Author	Significance* (Direction and magnitude)			
		
		Instrumental Research Utilization	Conceptual Research Utilization	Persuasive Research Utilization	Overall Research Utilization
**1. BELIEFS AND ATTITUDES**					

	Connor [66]	NS	NS	NS	S + (β = 0.234)
	
Attitude toward research	Estabrooks [32]	Canadian - S + (OR = 1.17) US Military - NS	**Not assessed**	**Not assessed**	Canadian - S + (OR = 1.21) US Military - S + (OR = 1.16)
	Kenny [63]	S + (β not reported)	NS	NS	NS
	
	Milner [67]	S + (β = 0.120)	NS	S + (β = 0.075)	S + (β = 0.098)

Importance of access to research	Kenny [63]	NS	NS	S + (β not reported)	NS

Cosmopoliteness	Milner [67]	NS	NS	NS	NS

Localite (orientation within one's immediate social context)	Milner [67]	NS	S + (β = 0.031)	NS	NS

Interest or organizational groups belonged to	Kenny [63]	NS	NS	NS	S + (β not reported)

Adoptiveness	Milner [67]	NS	NS	NS	NS

Belief suspension	Estabrooks [32]	Canadian - NS US Military - S + (OR = 1.11)	**Not assessed**	**Not assessed**	Canadian - S + (OR = 1.07) US Military - S + (OR = 1.08)
	Kenny [63]	S + (β not reported)	NS	NS	NS

	Connor [66]	NS	NS	NS	NS
	
Trust	Estabrooks [32]	NS	**Not assessed**	**Not assessed**	Canadian - NS US Military - S + (OR = 1.12)
	Kenny [63]	NS	NS	S + (β not reported)	NS

Research awareness	Milner [67]	S + (β = 0.037)	NS	S + (β = 0.076)	S + (β = 0.063)

Importance of various factors to decision-making	Kenny [63]	NS	S + (β not reported)	S + (β not reported)	NS

**2. INVOLVEMENT IN RESEARCH ACTIVITIES**					

Research involvement	Milner [67]	S + (β = 0.142)	NS	S + (β = 0.170)	S + (β = 0.176)

**3. INFORMATION SEEKING**					

Number of nursing journals read	Connor [66]	NS	NS	NS	NS
	Kenny [63]	NS	NS	NS	NS

Sources of knowledge	Connor [66]	NS	NS	NS	NS
	Estabrooks [32]	NS	**Not assessed**	**Not assessed**	NS
	Kenny [63]	NS	NS	NS	NS

Mass media	Milner [67]	NS	NS	S + (β = 0.194)	NS

Number of journals read	Kenny [63]	S + (β not reported)	NS	NS	NS

Number of continuing education sessions	Connor [66]	NS	S + (β not reported)	S + (β not reported)	S + (β not reported)

In-services attended	Connor [66]	NS	NS	NS	NS
	
	Estabrooks [32]	NS	**Not Assessed**	**Not Assessed**	Canadian - S + (OR = 1.03) US Military - NS

**4. EDUCATION**					

Increasing levels	Kenny [63]	NS	NS	NS	NS
	Connor [66]	NS	NS	NS	NS

Type of degree	Estabrooks [32]	NS	**Not Assessed**	**Not Assessed**	NS

Possessing a degree	Milner [67]	NS	NS	NS	NS

**5. PROFESSIONAL CHARACTERISTICS**					

Years employed as an RN	Estabrooks [32]	Canadian - NS US military - S + (OR = 0.97)	**Not Assessed**	**Not Assessed**	NS
	Kenny [63]	NS	NS	NS	NS

Length of time at job title	Connor [66]	NS	NS	NS	NS

Years in post (hospital)	Kenny [63]	NS	NS	NS	NS
	Connor [66]	NS	NS	NS	NS

Current role	Milner [67]^1^	NS	NS	NS	S - (β = -0.265)
	Milner [67]^2^	NS	S - (β = -0.382)	S - (β = -0.345)	NS
	Kenny [63]	NS	NS	NS	NS
	Connor [66]	NS	NS	NS	NS
	Kenny [63]	NS	NS	NS	NS
	Connor [66]	NS	NS	NS	NS

Number of memberships held	Connor [66]	NS	NS	NS	NS

**6. SOCIO-DEMOGRAPHIC AND SOCIO-ECONOMIC FACTORS**					

Age	Milner [67]	NS	NS	NS	S - (β = -0.011)
	
	Profetto-McGrath [64]	NS	NS	NS	NS

Gender	Connor [66]	NS	NS	NS	NS
	Estabrooks [32]	NS	**Not assessed**	**Not Assessed**	NS

**7. CRITICAL THINKING**					

Critical thinking skills (total CCTDI score)	Profetto-McGrath [64]	S + (*r *= .240	S + (*r *= .27)	S + (*r *= .17)	S + (*r *= .35)
	
	Profetto-McGrath [65]	S + (*r *= .222)	S + (*r *= .205)	S + (*r *= .237)	S + (*r *= .146)

*Significance: NS = not significant, S = significant at p < 0.05

^1^ Managers vs educators

^2^ RNs vs educators

We present the findings from this review update descriptively according to: the individual characteristics assessed, and whether research utilization was assessed as a general phenomenon or as specific kinds. We used the same six categories of individual nurse characteristics suggested in the earlier review by Estabrooks *et al*. (2003) for comparability: beliefs and attitudes, involvement in research activities, information seeking, education, professional characteristics, and other socioeconomic factors. A seventh category, critical thinking, emerged and is reported on in this review with respect to kinds of research utilization. Examples of the characteristics that fall within each of these categories can be seen in Tables 1 and 2.

We used a vote-counting approach to data synthesis. That is, the overall assessment of evidence for the association between an individual characteristic and research utilization was based on the relative number of studies demonstrating, and failing to demonstrate, statistically significant associations. As recommended by Grimshaw *et al*. [26], we supplemented this approach by also extracting all associations showing a positive direction of effect and the magnitude of effect for statistically significant effects (regardless of direction) when it was provided in the articles. These details are presented in Tables 1 and 2. However, because of large inconsistencies in how the associations were evaluated between studies, limited conclusions on the magnitude of the associations between research utilization and specific individual characteristics could be drawn.

We developed the following set of *a priori *rules to guide our synthesis:

1. In order to reach a conclusion as to whether or not an individual characteristic was associated with research utilization by nurses, it had to be assessed in a minimum of four articles. Characteristics assessed in less than four articles were coded as inconsistent (*i.e*., insufficient evidence to reach a conclusion). There is no agreed benchmark with respect to the number of studies required to reach a conclusion concerning the relationship between two or more variables when conducting a systematic review. Within the Cochrane Collaboration, where higher levels of evidence (*e.g*., randomized controlled trials, pseudo-randomized controlled trials) are routinely utilized, at least one high quality study is recommended; with more studies desired. When only lower levels of evidence (*e.g*., non-randomised studies, observational studies) are available, no direction with respect to the number of studies required is offered [27]. A recent review [28] (utilizing observational studies) that examined the extent to which social cognitive theories (that are comprised of individual characteristics) explain healthcare professionals' intention to adopt clinical behavior used a cut-off of three studies. In this review, we set our cut-off slightly higher, at four studies, to ensure we did not draw conclusions based on occasional/random findings.

2. Characteristics that were assessed in four or more articles were coded as significant, not significant, or equivocal, depending on which of these three categories 60% or more of the articles fell within. For example, if four articles existed and two of these articles found the characteristic to be significant and two articles not significant, the characteristic was coded as equivocal.

3. Where bivariate and multivariate statistics were both offered in an article as evidence, we used the more robust multivariate findings in our synthesis to reach a conclusion as to whether or not a relationship existed between the individual characteristic(s) and research utilization.

## Results

### Description of studies

**Figure 1:**
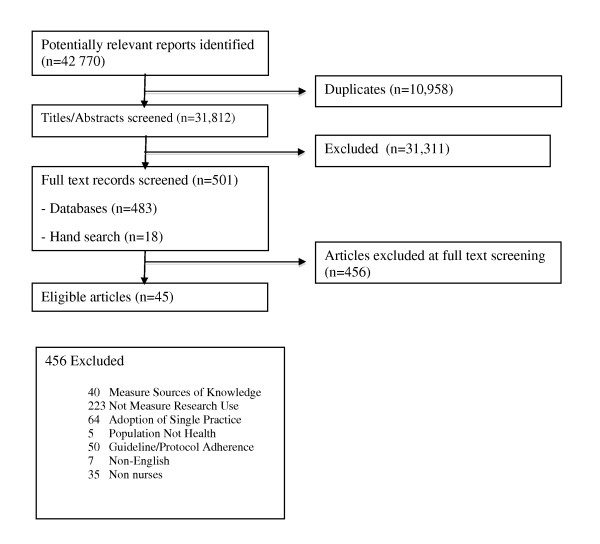
**Selection of articles for review**.

Figure 1 summarizes article selection for this review. The database and hand searches yielded 42,770 titles and abstracts. Of these 42,770 articles, 501 were identified as being potentially relevant after a title and abstract review. A total of 456 articles were excluded for not meeting our inclusion criteria, leaving 45 articles for inclusion in this review, and 31 (69%) of these articles are additions to the previous review). The 45 articles represent 41 original studies; four studies have two reports each: McCleary and Brown [29,30]; Estabrooks [31,32]; McCloskey [33,34]; and Parahoo [35,36]. A list of all (n = 45) included articles can be found in Additional File 4. The original review [17] included 22 articles. This review update excluded eight of these articles, leaving 14 of the original articles in the update. The eight articles were excluded for one of three reasons: they did not include a measure of research utilization as we defined it for this review update (n = 5) [37-41], they did not report on individual characteristics (n = 2, these two articles represented a second report of a study that did not report individual characteristics - the first report of each study, which did report on individual characteristics, were included) [2,42], or did not provide a quantitative (statistical) test of the association between the individual characteristic(s) and research utilization (n = 1) [43].

A variety of self-report instruments, multi-item and single item, were used to measure research utilization in the 45 included articles. Multi-item instruments used included: the Nurses Practice Questionnaire (n = 8) [12,14,44-49]; the Research Utilization Questionnaire (n = 11) [33,34,50-58]; the Edmonton Research Orientation Survey (n = 3) [29,30,59]; and three research utilization indexes, each used in a single study [60-62]. Single-item instruments used included: Estabrooks Kinds of Research Utilization Items (n = 9) [31,32,63-69]; Parahoo's Item (n = 2) [35,36]; Past, Present, and Future Use Items (n = 3) [70-72]; and other single items, each used in a single study (n = 6) [73-78]. The majority of articles examined research utilization by nurses in the United States (n = 18, 40%) followed by Canada (n = 14, 31%), Europe (n = 8, 18%), Australia (n = 2, 4.5%), China (n = 2, 4.5%), and Africa (n = 1, 2%). The most commonly reported setting was hospitals (n = 28, 62%) followed by a mixture of settings, *e.g*., sampling from a provincial or state nursing roster (n = 13, 29%), nursing homes (n = 2, 4.5%), an educational setting (n = 1, 2%), and a flight team setting (n = 1, 2%). With respect to year of publication, the vast majority of articles were published since 1995 (n = 40, 89%). Further details on the characteristics of the included articles can be found in Additional File 4.

### Methodological quality of included studies

Methodological quality of the articles included in this review is reported in Additional Files 2 and 3. All articles used an observational design: the majority (n = 43, 96%) used a cross-sectional design while two articles (4%) used a quasi-experimental design. Of the 45 included articles, one (2%) was rated as strong, 13 (29%) as moderate-strong, 18 (40%) as moderate-weak, and 13 (29%) as weak. Discrepancies in quality assessment related mainly to sample representativeness, treatment of missing data, and appropriateness of the statistical test(s) used.

### The outcome: individual characteristics and research utilization

Data on individual characteristics were extracted into the original six categories from the previous review [17]: beliefs and attitudes, involvement in research activities, information seeking, education, professional characteristics, socio-demographic and socio-economic factors (relabeled from other socio-economic factors), and one additional category, and critical thinking. Relationships between these characteristics and research utilization in general, and kinds of research utilization, are summarized next with additional details presented in Tables 1 and 2 respectively.

### Research utilization in general

A total of 39 (87%) articles examined relationships between individual characteristics and nurses' research utilization in general (Table 1).

### Beliefs and attitudes

Fourteen articles assessed one or more individual characteristic in the beliefs and attitudes category. Of these 14 articles, six were rated as weak methodologically, five were rated as moderate-weak, and three were rated as moderate-strong (Additional Files 2 and 3). Sample sizes varied from a low of 20 participants [54] to a high of 1,117 participants [62] (Additional File 4). The most frequently assessed characteristic in this category was attitude towards research, assessed in eight articles. The majority of these eight articles were rated as weak (n = 3) or moderate-weak (n = 4) methodologically while one article received a quality rating of moderate-strong (Additional Files 2 and 3). In all eight articles, attitude towards research was measured using multi-item summated scales. A 21-item scale developed by Champion and Leach [50] with items tapping nurses' feelings about incorporating research into practice was used in four of the eight articles [50,52,56,57]. Similar multi-item measures, with six [31,61], 12 [54] and 15 items [72] were used in the remaining four studies. A positive association with research utilization, at statistically significant levels, was found in all eight articles. The magnitude of effect, on average, was high moderate, with correlation coefficients ranging from 0.41 to 0.82. Other belief and attitudinal characteristics were assessed in less than four articles and therefore their results cannot be considered with any confidence.

### Involvement in research activities

Thirteen articles assessed one or more individual characteristic related to nurses' involvement in research activities. Of these articles, three were rated as weak methodologically, eight were rated as moderate-weak, and two were rated as moderate-strong (Additional Files 2 and 3). Sample sizes also varied from a low of 82 participants [55] to a high of 1,100 participants [49] (Additional File 4). Examples of activities assessed that were reflective of involvement in research activities included: participation in a research study [44,55], participation in quality improvement initiatives [58], participation in quality management [29], and data collection for others conducting research [71]. Additional examples can be found in Table 1. A total of 13 individual characteristics were identified in this category overall. However, each characteristic was assessed in less than four articles, precluding us from drawing conclusions on the relationships between individual characteristics typical of involvement in research activities and nurses' use of research findings in practice.

### Information-seeking

A total of 15 articles reported individual characteristics consistent with information-seeking behavior. Two articles were rated as weak methodologically, five articles as moderate-weak, and the remaining eight articles as moderate-strong (Additional Files 2 and 3). Sample sizes varied largely from a low of 92 participants [57] to a high of 5,948 participants [69] (Additional File 4). Several articles examined the relationships between different reading practices and research utilization. For example, reading professional journals [46]; hours spent reading professional journals [44,47,48]; the number of journals read [12,49,72]; and reading specific journals such as *Heart and Lung *[47,48], *Nursing Research *[44,48], and *RN *[44], were studied. Different combinations of these six reading characteristics were tested a total of 12 times (some articles assessed more than one of the reading practices simultaneously). Findings from these investigations were equivocal with seven articles (58%) reporting statistically significant findings and five articles (42%) not finding statistically significant findings. Thus, no conclusion can be drawn as to the effect of reading practices on nurses' use of research in practice.

The second most commonly studied information-seeking characteristic was attendance at conferences and/or attendance at in-services, examined in five articles [31,47-49,71]. Four of these articles [31,47-49], all rated moderate-strong with respect to methodological quality, found positive relationships, at statistically significant levels, between conference and/or in-service attendance and research utilization. The overall magnitude of this effect, however, is not computable since each article used a different test of statistical association. The remainder of individual characteristics falling within the category of information seeking were only investigated in one or two articles, precluding us from considering their findings (Table 1).

### Education

A total of 28 articles reported individual characteristics within the domain of education, making it the most commonly studied category of characteristics in this review. Of the 28 articles, 10 were rated as weak methodologically, nine were rated as moderate-weak, and nine were rated as moderate-strong (Additional Files 2 and 3). Sample sizes varied from a low of 20 participants [54] to a high of 5,948 participants [69] (Additional File 4).

Twenty-five of the articles in this category examined one of three characteristics related to formal nursing education: increasing levels of education (*i.e*., diploma, bachelor degree, masters degree, PhD degree, but without *post hoc *analyses to determine between which levels noted differences lied), type of degree: bachelor versus diploma, and type of degree: graduate degree (masters or PhD) versus lower (bachelor and/or diploma). Increasing levels of education was assessed in seven articles [12,44,47,49,54,55,77]. Findings from these investigations were equivocal with only four (57%) of these articles [12,49,54,77] finding positive relationships, at statistically significant levels, between higher levels of education and research utilization. A total of 11 articles [14,29,31,46,57,61,62,69,71,73,76] examined the relationship between research utilization and type of degree: bachelor versus diploma. Eight of these articles [14,31,46,57,61,62,73,76] did not find a significant association between bachelor degree versus diploma and research utilization, leading to the conclusion that type of degree: bachelor versus diploma is not an important characteristic to nurses' use of research. An additional seven articles (six studies) [29,33,34,48,50,59,60] examined the relationship between research utilization and type of degree: graduate degree (masters or PhD) versus lower (bachelor and/or diploma). The majority of these articles (n = 5, 71%) found a statistically significant relationship between graduate degree versus bachelor degree/diploma and research utilization [29,33,34,48,50,59]. Overall, findings from all 25 articles examining characteristics related to formal nursing education levels indicate that a positive effect exists for level of education, when a nurse holds a graduate degree compared to a bachelor degree/diploma but not when a nurse holds a bachelor degree compared to a diploma.

Another educational characteristic assessed in greater than four articles was completion of research classes [12,30,44,47,55]. Findings showed that this characteristic however was not significantly related to research utilization. Two articles [12,30], rated as weak and moderate-strong methodologically respectively, found a positive relationship, at statistically significant levels, while three articles (60%) [44,47,55], one rated as weak methodologically and two rated as moderate-strong, did not find evidence of a statistically significant relationship.

The remaining individual characteristics related to education (*e.g*., well prepared in education process, working towards a degree, number of degrees, see Table 1) were assessed in less than four articles and therefore, were not considered.

### Professional characteristics

The second most commonly studied category of individual characteristics, assessed in 27 of the 39 included articles, was professional characteristics. Of these articles, 12 were rated as weak methodologically, eight as moderate-weak, and eight as moderate-strong (Additional Files 2 and 3). Sample sizes varied from a low of 20 participants [54] to a high of 5,948 participants [69] (Additional File 4).The most commonly reported characteristics in this category were: experience (*i.e*., years employed as a nurse) (n = 12 articles), current role (*e.g*., leadership compared to staff nurse) (n = 10 articles), clinical specialty (*e.g*., critical care compared to medical/surgical (n = 9 articles) and job satisfaction (n = 5 articles) (Table 1). Of these characteristics, consistent statistically significant relationships with research utilization were found for current role, specialty, and job satisfaction. Experience was not related to research utilization.

Ten articles (nine studies) examined the impact of current role on research utilization. Six (60%) of these articles (three rated as weak methodologically, two as moderate-weak, and one as moderate- strong, see Additional Files 2 and 3) found that nurses practicing in advanced practice or leadership roles had significantly higher research utilization scores compared to staff nurses [33,34,52,59,69,71]. However, nurses in such advanced practice and leadership roles generally have higher levels of education, which may have confounded this finding. Nine articles examined the impact of clinical specialty on research utilization. Six (67%) of these articles (two rated as weak, as moderate-weak, and as moderate-strong respectively, see Additional File 2) found a significant relationship between specialty and research utilization; nurses who worked on specialty wards (*e.g*., critical care, diabetes care) reported higher frequencies of research utilization in comparison to nurses who worked in more generalized units (*e.g*., medical or surgical floors) [14,36,53,55,60,62]. Five articles examined the impact of job satisfaction on research utilization. Three (60%) of these articles (one rated as moderate-weak methodologically and two as moderate-strong, see Additional File 2) found a statistically significant relationship between job satisfaction and research utilization [47,62,69]. Experience, assessed in 12 articles, was not related to research utilization at statistically significant levels in the majority (n = 10 of 12, 83%) of these articles (Table 1).

### Socio-demographic and socio-economic factors

Of the ten articles reporting other socio-demographic and socio-economic nurse characteristics (four rated as weak methodologically, three as moderate-weak, and three as moderate-strong, see Additional File 2), none reported a significant association with research utilization. Further, with the exception of age, which was assessed in nine studies, the characteristics were assessed in less than four studies, precluding the drawing of conclusions.

### Kinds of research utilization

While the majority of articles identified in this review update assessed associations between individual characteristics and nurses' use of research in general, there is also a beginning trend in the literature to examine kinds of research utilization. A total of six articles (one rated as weak methodologically, two as moderate-weak, two as moderate-strong, and one as strong, see Additional File 2) were identified that explicitly examined the relationship between individual characteristics and nurses' use of one or more kinds of research utilization. The following section presents an overview of the findings from these six articles. More details on these findings can be found in Table 2.

The only individual characteristic assessed in a sufficient number of articles (*i.e*., in four or more articles) was a nurse's attitude towards research. All four articles reported a positive relationship, at statistically significant levels, between a nurse's attitude towards research and at least one kind of research utilization [32,63,66,67]. Only instrumental and overall kinds of research utilization were assessed in four articles. A positive relationship was found in three articles (75%) for both of these kinds of research utilization: instrumental [32,63,67] and overall [32,66,67]. All remaining characteristics were assessed in less than four articles, precluding conclusions.

One individual characteristic, critical thinking dispositions, was assessed in two articles examining kinds of research utilization. Critical thinking dispositions refers to a "set of attitudes that define a personal disposition to prize and to use critical thinking in one's personal, professional, and civic affairs" [79]. Both articles assessed critical thinking dispositions using the California Critical Thinking Disposition Inventory that measures seven dispositional components: truth-seeking, open-mindedness, analyticity, systematicity, self-confidence, inquisitiveness, and maturity [79]. Both identified studies found a positive relationship, at statistically significant levels, between nurses' ability to think critically (as measured by an average of all seven dispositions) and each of the four kinds of research utilization [64,65]. The magnitude of this effect was small to moderate with correlation coefficients ranging from 0.15 to 0.35, depending on the kind of research utilization (Table 2).

## Discussion

### Comparison with previous review

This systematic review update focused on individual nurse characteristics that have been studied empirically with respect to nurses' use of research in practice. By extending the search criteria of the previous review, 31 additional studies were identified for inclusion in this update. This more than doubles the evidence available for review specifically examining the relationships between individual characteristics and research utilization by nurses. Unfortunately, studies continue to vary greatly in terms of sample selection (source of participants), sample size, study methods and rigor, statistical tests used, and the instrument (items) used to measure the outcome variable -- research utilization. Promisingly, though, a trend in the most recent included studies for more robust analyses (*i.e*., multivariate regression versus bivariate correlations and/or tests of difference) and less variability in choice of outcome measures is evident. Nevertheless, given the continuing heterogeneity between studies, only general statements can be made regarding the relationships between individual characteristics and research utilization by nurses at this time. That is, at this point in time we can only say which characteristics are associated with research utilization and not which characteristics predict research utilization by nurses.

Taken collectively, the now significantly larger body of evidence suggests promise for the following individual characteristics as being important to (*i.e*., related to an increase in) nurses' use of research in their practice: positive attitude towards research, attending conferences and/or in-services, having a graduate degree (compared to a bachelors degree or diploma), current role (*i.e*., leadership and/or advanced practice compared to staff nurse), clinical specialty (working in critical care areas compared to general hospital units), and job satisfaction. An additional three characteristics were shown not to be important to research utilization by nurses: completion of research classes, experience, and age. While, overall, the extent to which many individual characteristics influence research utilization remains largely unknown, there is support for the above-mentioned characteristics. This represents a significant increase in knowledge over the previous review. Table 3 compares conclusions made in our review update with the original review.

**Table 3:** Comparison of conclusion between previous review and review update

Category	Individual Characteristic	Conclusion	
		
		Previous Review (Estabrooks *et al*., 2003	Review Update
Beliefs and Attitudes	Attitude towards research	Positive attitude associated with more research use	Positive attitude associated with more research use (in general **and with instrumental and overall research utilization**)
	
	All other determinants	No conclusion - Too few studies	No conclusion - Too few studies

Involvement in Research Activities	Variety of determinants	No conclusion - Too few studies	No conclusion - Too few studies

	Reading practices	Equivocal	Equivocal
	
Information Seeking	Attending conferences/in-services	No conclusion - Too few studies	**Conference and/or in-service attendance associated with more research use**
	
	All other determinants	No conclusion - Too few studies	No conclusion - Too few studies

Education	Type of Degree	Equivocal	**Bachelors versus diploma - no effect on research use**
			
			**Graduate versus bachelors/diploma - increased research use for graduate degree**
	
	Completion of research classes	No conclusion - Too few studies	**No association with research use**
	
	All other determinants	No conclusion - Too few studies	No conclusion - Too few studies

Professional Characteristics	Years as an RN	No association with research use	No association with research use
	
	Current role	Leadership role associated with more research use	Leadership role associated with more research use
	
	Clinical specialty	No association with research use	**Working in critical care areas (compared to general wards) associated with more research use**
	
	Job satisfaction	No conclusion - Too few studies	**Higher levels of job satisfaction associated with more research use**
	
	All other determinants	No conclusion - Too few studies	No conclusion - Too few studies

Socio-Demographic and Socio-Economic Factors	Age	No association with research use	No association with research use
	
	All other determinants	No conclusion - Too few studies	No conclusion - Too few studies

### Kinds of research utilization

In addition to examining the relationships between individual characteristics and research utilization generally, we also looked for relationships between individual characteristics and kinds (*i.e*., instrumental, conceptual, persuasive, and overall) of research utilization. Estabrooks [2] confirmed the existence of the four kinds of research utilization in a study of Canadian registered nurses, and additional studies since then have shown differential relationships between individual and contextual characteristics and the different kinds of research utilization [32,63,66,67]. Therefore, we elected to report on these articles separately and not combine them with the articles that report on research utilization in general. While few articles were identified that have assessed relationships between individual characteristics and kinds of research utilization, some promising findings did emerge in those that were identified. For example, critical thinking, which was assessed in two articles showed positive, statistically significant correlations with each kind of research utilization in both articles [64,65]. These two articles were moderate-weak and moderate-high in methodological quality and had relatively small sample sizes of 143 and 287 nurses, respectively (Additional Files 2 and 4). This, combined with the limited number of studies conducted, precluded us from drawing a conclusion. While there is insufficient evidence at this time to conclude that a relationship does exist (and that nurses' critical thinking dispositions could be a target of future intervention studies), it may be a fruitful avenue for future research.

Despite a limited number of articles addressing kinds of research utilization, one characteristic -- attitude towards research -- was assessed in a sufficient number of articles (*i.e*., four articles) to be able to conclude a positive relationship between attitude towards research and nurses' instrumental and overall use of research exists. This relationship was also found in all eight articles examining attitude towards research on research utilization in general. This finding is consistent with known theories of human behavior. For example, the Theory of Planned Behavior, which is frequently used in psychological research, states human behavior (such as research utilization) is guided by three kinds of considerations: behavioral beliefs (*i.e*., beliefs about the likely outcomes of a behavior), normative beliefs (*i.e*., beliefs about the normative expectations of others and motivation to comply with these expectations), and control beliefs (*i.e*., beliefs about the presence of factors that may facilitate or impede performing the behavior) [80]. Behavioral beliefs are further known to produce a favorable or unfavorable attitude toward the behavior [80], supporting our findings.

Godin *et al*. [28], in a systematic review of healthcare professionals' (that included nurses) intentions and clinical behaviors, found the Theory of Planned Behavior to be an appropriate theory for examining attitudes and beliefs in relation to specific actions or behaviors. Specifically, they found healthcare professionals' beliefs about their own capabilities and the consequences of their behavior to be consistently and positively associated, at statistically significant levels, with predicting their clinical behavior. Beliefs were also positively and significantly associated with healthcare professionals' intention to change their behavior. These findings illustrate the potential benefit that using this theory, beyond the measurement of nurses' attitudes in general towards research utilization, may have in research utilization studies. For example, added value could be obtained by measuring nurses' beliefs and attitudes in relation to specific behaviors (*i.e*., their use of specific research-based findings in practice). Future research should also focus on determining what causes nurses to form favorable (positive) attitudes towards the use of research, both of research utilization in general and of its kinds, as well as of the use of specific research-based findings in practice.

### Methodological implications for future research

Systematic reviews typically identify and comment on problems with internal validity of the research under scrutiny, and this review update is no exception. Future studies examining individual characteristics related to research utilization need to attend to methodological quality to reduce bias and to increase confidence in this growing body of knowledge. This will allow for the design of theory-informed research utilization interventions with the intention of improving the quality of patient care.

Four important limitations of studies conducted to date on individual characteristics and research utilization by nurses are: methodological quality, statistical rigor, inconsistency in measurement of the outcome measure (research utilization), and limited use of research utilization or other related theory. First, few studies examining the relationship between individual characteristics and research utilization in this review were of moderate-strong or strong methodological quality, illustrating a clear need for well-designed, robust studies that examine the association between different individual characteristics and research utilization by nurses. Second, in order to effectively design research utilization interventions tailored for individual nurse characteristics, we need to know which characteristics predict (not just which ones are related to) research utilization. This will require multivariate statistical assessments. There is no need for continued bivariate assessments, especially given the clear evidence of inter-correlations among different individual characteristics [81]. Third, there is inconsistency in the measures being used for the outcome of interest, research utilization. By this we mean that we observed a lack of standard measures of research utilization across studies. While a few instruments that measure research use by nurses have been used in multiple studies (*e.g*., Nurses Practice Questionnaire [12,14,44-49], Research utilization Questionnaire [33,34,50-58]), Edmonton Research Orientation Survey [29,30,59], Estabrooks Kinds of Research Utilization Items [31,32,63-69]), by far, the most common approach to measuring research utilization has been the use of a single-item developed for an individual study. This absence of commonly used measures across studies makes it difficult, if not impossible, to build a consistent body of knowledge on which individual characteristics influence research utilization by nurses. Finally, only one-third (n = 14) of the articles identified in this review reported their investigation was based on research utilization or other appropriate theory (Additional File 4). For the vast majority of these articles, Rogers Diffusion of Innovations theory was used to guide the development of a measure of and/or calculation of a research utilization score, but not the selection of variables of included or the design and evaluation of the study. Future research utilization investigations should utilize appropriate theory in both instrument and study design/evaluation.

### Limitations

While rigorous methods were used for this review, there were limitations. First, while an attempt was made to review grey literature (*e.g*., searching dissertation databases) we did not search all grey literature databases, and, as such, this review update may not be representative of all relevant work in the field. Second, where details of study methods were not clear, we did not attempt to clarify these details by contacting the article authors. This may have resulted in aspects of methods being scored low in the quality assessment phase, possibly reflecting quality of the reporting rather than the actual methods used. Third, studies published in languages other than those of the research team were excluded. Finally, because of the inconsistency in how associations between individual characteristics and research utilization were determined and reported in the included studies, we were forced to use a vote-counting approach to data synthesis. There are several weaknesses associated with using vote counting. For example, this approach to synthesis fails to account for: effect sizes (vote counting gives equal weight to all associations, regardless of magnitude) and precision of the estimate from the primary studies (vote counting gives equal weight to comparisons irrespective of sample size). To lessen these problems, we reported the following as recommended by Grimshaw *et al*. [26]: all associations showing a positive direction of effect, the number of comparisons showing statistically significant effects (regardless of direction), and the magnitude of effect for significant findings when it was provided in the articles.

## Conclusion

This review update points to an increased body of research on the study of individual characteristics and research utilization by nurses. However, methodological problems inherent in many of the studies included in the review update mean that robust evidence to support individual characteristics that predict research utilization is scarce. Current evidence suggests that a nurse's attitude towards research is the only individual characteristic that is consistently (with a positive effect) related to research utilization in general and the different kinds of research utilization. Other individual characteristics with evidence for a positive association with research utilization (in general) include: attending conferences and/or in-services, having a graduate degree, current role, clinical specialty, and job satisfaction. These characteristics may hold promise as targets of future research utilization interventions. While all of these characteristics are potentially modifiable, some can be more easily manipulated and thus incorporated into interventions to increase research utilization. For example, attitude towards research and attendance at conferences and/or in-services are two characteristics that we believe can and should be the focus of future research utilization interventions. The remaining characteristics identified in this review as having a positive statistically significant association with research utilization, while modifiable, would require more effort and time to implement (e.g., increasing the number of nurses employed within a clinical setting that hold a graduate degree).

We also recommend that programmatic research in the area of research utilization in nursing be undertaken. Programmatic research differs from conducting a research study in that it seeks to break a large research topic into smaller, more manageable pieces, allowing for more detailed analyses. Importantly, programmatic research addresses each piece sequentially in an effort to build a coherent picture from the smaller studies' findings, and allows investigators to build upon their own and others' research. Such programs in research utilization in nursing would have several concurrent streams examining, for example, different settings (acute care adults, acute care pediatrics, long-term care, community/home healthcare), different classes of determinants (individual characteristics, contextual factors, and organizational factors), and interventions to increase research use and subsequently patient outcomes. Without such programmatic research, we believe substantial advances in understanding how to increase the use of research by nurses and thereby improve patient care will be difficult, if not impossible, to achieve.

## Competing interests

The authors declare that they have no competing interests.

## Authors' contributions

All authors participated in designing the study, securing funding for the project, and developing the search strategy. JES undertook the article selection; data extraction and quality assessment; and drafted the manuscript. CAE, PG, and LW provided valuable advice throughout the study. All authors provided critical commentary on the manuscript and approved the final version.

## Supplementary Material

Additional file 1 **Search strategy**. A summary of the search strategy used in the review. Click here for file

Additional file 2 **Quality assessment for included cross sectional articles**. A description of the findings from the quality assessment of included articles describing studies that used a cross sectional study design. Click here for file

Additional file 3 **Quality assessment for the included quasi-experimental articles**. A description of the findings from the quality assessment of included articles describing studies that used a quasi-experimental study design. Click here for file

Additional file 4 **Characteristics of the included studies**. A detailed summary of the characteristics of all articles included in the review. Click here for file
