# Supplementary material for: Individual determinants of research utilization by nurses: a systematic review update
Source: Implement Sci. 2011 Jan 5;6:1. doi: 10.1186/1748-5908-6-1 (PMC3024963; doi:10.1186/1748-5908-6-1)
Supplement: Additional file 1 — Search strategy. A summary of the search strategy used in the review. [file 1748-5908-6-1-S1.DOC]

**Additional File 1. Search strategy**

The following bibliographic databases were searched: Cochrane Database of Systematic Reviews (through to Fourth Quarter 2008), Cochrane Central Register of Controlled Trials (CENTRAL) (through to Fourth Quarter 2008); Health and Psychosocial Instruments (HAPI) (through to 14 October 2008), MEDLINE (through to 11 October 2008), CINAHL (through to 11 October 2008), EMBASE (through to 13 October 2008), Web of Science (through to 13 October 2008), SCOPUS (through to 17 November 2008), OCLC Papers First (through to 13 October 2008), OCLC WorldCat (through to 13 October 2008), Sociological Abstracts (through to 13 October 2008), Proquest Dissertation Abstracts (through to 14 October 2008), and Proquest ABI Inform (through to 14 October 2008).

The search terms used were as follows:

"nursing practice questionnaire" or "edmonton research orientation survey" or (technolog* W diffus*) or (chnolog* W transfer*) or (technolog* W translat* ) or (technolog* W adopt*) or "diffusion of innovation" or "diffusion of innovations" or "innovation diffusion" or "dissemination of innovation" or "dissemination of innovations" or (innovation* W/1 adopt*) or "adoption of innovation" or "adoption of innovations" or "dissemination of evidence" or "implementation of evidence" or "adoption of evidence" or "uptake of evidence" or "use of evidence" or "utilization of evidence" or "utilisation of evidence" or "diffusion of evidence” or "translation of knowledge" or "transfer of knowledge" or "implementation of knowledge" or "adoption of knowledge" or "uptake of knowledge" or "utilization of knowledge" or "utilisation of knowledge" or "dissemination of knowledge" or "diffusion of knowledge" or "implementation of technologies" or "adoption of technologies" or "uptake of technologies" or "dissemination of technologies" or "diffusion of technologies" or "translation of technologies" or "transfer of technologies" or "implementation of technology" or "adoption of technology" or "uptake of technology" or "dissemination of technology" or "diffusion of technology" or "translation of technology" or "transfer of technology" or "translation of research" or "transfer of research" or "implementation of research" or "adoption of research" or "uptake of research" or "use of research" or "utilization of research" or "utilisation of research" or "dissemination of research" or "diffusion of research" or "evidence uptake" or "evidence use" or "evidence diffusion" or "evidence dissemination" or "evidence utilization" or "evidence utilisation" or "evidence transfer" or "evidence translation" or "evidence implementation" or "evidence adoption" or "knowledge uptake" or "knowledge use" or "knowledge diffusion” or "knowledge dissemination" or "knowledge utilization" or "knowledge utilisation" or "knowledge transfer" or "knowledge translation" or “knowledge implementation" or "knowledge adoption" or “research uptake" or "research use" or "research diffusion" or "research dissemination" or "research utilization" or "research utilisation" or "research transfer" or "research translation" or "research implementation" or "research adoption" **AND** survey* or questionnaire* or inventor* or instrument* or scale* or assess* or evaluat* or measur* or tool* or reliability or validity or validation or reproducib* or benchmark* or psychometric
