# Supplementary material for: Individual determinants of research utilization by nurses: a systematic review update
Source: Implement Sci. 2011 Jan 5;6:1. doi: 10.1186/1748-5908-6-1 (PMC3024963; doi:10.1186/1748-5908-6-1)
Supplement: Additional file 2 — Quality assessment for included cross sectional articles. A description of the findings from the quality assessment of included articles describing studies that used a cross sectional study design. [file 1748-5908-6-1-S2.DOC]

**Additional File 2. Quality assessment for included cross sectional articles (n =** 43)

| **First Author, Year** | **Sample** | | | | | | | | | | | | | **Measurement** | | | | | | | **Statistical Analysis** | | | | | **Total Points^1^** | **Score** | **Quality** |
| --- | --- | --- | --- | --- | --- | --- | --- | --- | --- | --- | --- | --- | --- | --- | --- | --- | --- | --- | --- | --- | --- | --- | --- | --- | --- | --- | --- | --- |
|  | **Probabilistic sample used** | **Representative** | | **Sample size appropriate for power** | | **Sample drawn > 1 site** | | **Matching design** | | **Statistically adjusted** | | **Response rate > 50%** | | **DV directly measured/administrative** | | **DV reliability** | | | **DV validity** | | **Appropriate tests used** | | **p values reported** | **CI reported** | **Missing data managed appropriately** |  |  |  |
| Milner, 2005 | 1 | 2 | | 1 | | 1 | | 2 | | N/A | | 1 | | 0 | | 1 | | | 1 | | 1 | | 1 | 0 | 1 | 13/15 | 0.87 | Strong |
| Rodgers, 2000 | 1 | 2 | | 1 | | 1 | | N/A | | N/A | | 1 | | 0 | | 1 | | 1 | | | 1 | | 1 | 0 | 1 | 11/13 | 0.85 | Moderate-Strong |
| Squires, 2007 | 0 | 1 | | 1 | | 1 | | N/A | | N/A | | 1 | | 0 | | 1 | | 1 | | | 1 | | 1 | 1 | 1 | 10/13 | 0.77 | Moderate-Strong |
| Coyle, 1990 | 1 | 2 | | 1 | | 1 | | N/A | | N/A | | 1 | | 0 | | 1 | | 1 | | | 1 | | 1 | 0 | 0 | 10/13 | 0.77 | Moderate-Strong |
| Brett, 1987 | 1 | 2 | | 1 | | 1 | | N/A | | N/A | | 1 | | 0 | | 1 | | 1 | | | 1 | | 1 | 0 | 0 | 10/13 | 0.77 | Moderate-Strong |
| Barta, 1995 | 0 | 1 | | 1 | | 1 | | N/A | | N/A | | 1 | | 0 | | 1 | | 1 | | | 1 | | 1 | N/A | 1 | 9/12 | 0.75 | Moderate-Strong |
| Tsai, 2000 | 1 | 2 | | 1 | | 0 | | N/A | | N/A | | 1 | | 0 | | 1 | | 1 | | | 1 | | 1 | N/A | 0 | 9/12 | 0.75 | Moderate-Strong |
| Estabrooks, 2007 | 1 | 2 | | 1 | | 1 | | 0 | | 1 | | 0 | | 0 | | 1 | | 1 | | | 1 | | 1 | 1 | 1 | 12/16 | 0.75 | Moderate-Strong |
| Profetto-McGrath, 2009 | 1 | 2 | | 1 | | 1 | | N/A | | N/A | | 1 | | 0 | | 0 | | 1 | | | 1 | | 1 | N/A | 0 | 9/12 | 0.75 | Moderate-Strong |
| Estabrooks, 1999 | 1 | 2 | | 1 | | 1 | | N/A | | N/A | | 0 | | 0 | | 0 | | 1 | | | 1 | | 1 | 0 | 1 | 9/13 | 0.69 | Moderate-Strong |
| Michel, 1995 | 1 | 1 | | 1 | | 1 | | N/A | | N/A | | 1 | | 0 | | 1 | | 1 | | | 1 | | 1 | 0 | 0 | 9/13 | 0.69 | Moderate-Strong |
| Forbes, 1997 | 0 | 1 | | 1 | | 1 | | N/A | | N/A | | 0 | | 0 | | 1 | | 1 | | | 1 | | 1 | N/A | 1 | 8/12 | 0.67 | Moderate-Strong |
| Cummings, 2007 | 1 | 1 | | 1 | | 1 | | N/A | | N/A | | 0 | | 0 | | 0 | | 1 | | | 1 | | 1 | N/A | 1 | 8/12 | 0.67 | Moderate-Strong |
| Bonner, 2008 | 0 | 1 | | 1 | | 1 | | N/A | | N/A | | 0 | | 0 | | 1 | | 1 | | | 1 | | 1 | N/A | 1 | 8/12 | 0.67 | Moderate-Strong |
| Berggren, 1996 | 0 | 1 | | 1 | 1 | | N/A | | N/A | | 1 | | 0 | | | 1 | 1 | | | 1 | | N/A | | N/A | 0 | 7/11 | 0.64 | Moderate-Weak |
| Ofi, 2008 | 0 | 1 | | 1 | 1 | | N/A | | N/A | | 1 | | 0 | | | 0 | 1 | | | 1 | | 1 | | 1 | 0 | 8/13 | 0.62 | Moderate-Weak |
| Hatcher, 1997 | 1 | 2 | | 1 | 0 | | N/A | | N/A | | 0 | | 0 | | | 1 | 1 | | | 1 | | 1 | | 0 | 0 | 8/13 | 0.62 | Moderate-Weak |
| Prin, 1997 | 0 | 1 | | 1 | 0 | | N/A | | N/A | | 0 | | 0 | | | 1 | 1 | | | 1 | | 1 | | 0 | 1 | 8/13 | 0.62 | Moderate-Weak |
| Kenny, 2005 | 0 | 1 | | 1 | 1 | | N/A | | N/A | | 0 | | 0 | | | 1 | 1 | | | 1 | | 1 | | 0 | 1 | 8/13 | 0.62 | Moderate-Weak |
| Varcoe, 1995 | 1 | 1 | | 1 | 1 | | N/A | | N/A | | 0 | | 0 | | | 1 | 1 | | | 1 | | 1 | | 0 | 0 | 8/13 | 0.62 | Moderate-Weak |
| Logsdon, 1998 | 1 | 1 | | 1 | 1 | | N/A | | N/A | | 0 | | 0 | | | 0 | 1 | | | 1 | | 1 | | N/A | 0 | 7/12 | 0.58 | Moderate-Weak |
| Bostrom, 2008 | 0 | 1 | | 1 | 1 | | N/A | | N/A | | 1 | | 0 | | | 1 | 0 | | | 1 | | 1 | | N/A | 0 | 7/12 | 0.58 | Moderate-Weak |
| Humphris, 1999 | 1 | 2 | | 1 | 1 | | 0 | | 0 | | 1 | | 0 | | | 0 | 1 | | | 1 | | 1 | | 0 | 0 | 9/16 | 0.57 | Moderate-Weak |
| Brown, 1997 | 0 | 1 | | 1 | 1 | | N/A | | N/A | | 1 | | 0 | | | 0 | 1 | | | 1 | | 1 | | 0 | 0 | 7/13 | 0.54 | Moderate-Weak |
| McCleary, 2002 | 0 | 1 | | 1 | 0 | | N/A | | N/A | | 0 | | 0 | | | 1 | 1 | | | 1 | | 1 | | 0 | 1 | 7/13 | 0.54 | Moderate-Weak |
| Parahoo, 1999 | 0 | 1 | | 1 | 1 | | N/A | | N/A | | 1 | | 0 | | | 0 | 1 | | | 1 | | 1 | | 0 | 0 | 7/13 | 0.54 | Moderate-Weak |
| Parahoo, 2001 | 0 | 1 | | 1 | 1 | | N/A | | N/A | | 1 | | 0 | | | 0 | 1 | | | 1 | | 1 | | 0 | 0 | 7/13 | 0.54 | Moderate-Weak |
| Rutledge, 1996 | 0 | 1 | | 1 | 1 | | N/A | | N/A | | 0 | | 0 | | | 1 | 1 | | | 1 | | 1 | | 0 | 0 | 7/13 | 0.54 | Moderate-Weak |
| Wallin, 2003 | 0 | 2 | | 0 | 0 | | N/A | | N/A | | 1 | | 0 | | | 1 | 1 | | | 1 | | 1 | | 0 | 0 | 7/13 | 0.54 | Moderate-Weak |
| Wallin, 2006 | 0 | 1 | | 1 | 1 | | N/A | | N/A | | 1 | | 0 | | | 0 | 1 | | | 1 | | 1 | | 0 | 0 | 7/13 | 0.54 | Moderate-Weak |
| Profetto-McGrath, 2003 | 0 | 1 | | 1 | 1 | | N/A | | N/A | | 0 | | 0 | | | 1 | 1 | | | 1 | | 1 | | 0 | 0 | 7/13 | 0.54 | Moderate-Weak |
| McCleary, 2003 | 0 | 1 | 1 | | 0 | | N/A | | N/A | | 0 | | 0 | | 1 | | 1 | | | 1 | | 1 | | 0 | N/A | 6/12 | 0.5 | Weak |
| Erler, 2000 | 1 | 1 | 1 | | 1 | | N/A | | N/A | | 0 | | 0 | | 0 | | 0 | | | 1 | | 1 | | N/A | 0 | 6/12 | 0.50 | Weak |
| Stiefel, 1996 | 1 | 1 | 1 | | 1 | | 0 | | 0 | | 0 | | 0 | | 1 | | 1 | | | 1 | | 1 | | 0 | 0 | 8/16 | 0.5 | Weak |
| Champion, 1989 | 0 | 1 | 1 | | 0 | | N/A | | N/A | | 0 | | 0 | | 1 | | 1 | | | 1 | | 1 | | 0 | 0 | 6/13 | 0.46 | Weak |
| Lacey, 1994 | 1 | 1 | 0 | | 1 | | N/A | | N/A | | 0 | | 0 | | 0 | | 1 | | | 1 | | 1 | | 0 | 0 | 6/13 | 0.46 | Weak |
| Nash, 2005 | 1 | 0 | 1 | | 1 | | N/A | | N/A | | 0 | | 0 | | 1 | | 1 | | | 1 | | 0 | | 0 | 0 | 6/13 | 0.46 | Weak |
| Wells, 1994 | 1 | 2 | 1 | | 0 | | N/A | | N/A | | 0 | | 0 | | 0 | | 0 | | | 1 | | 1 | | 0 | 0 | 6/13 | 0.46 | Weak |
| McCloskey, 2005 | 0 | 0 | 1 | | 1 | | N/A | | N/A | | 0 | | 0 | | 1 | | 1 | | | 1 | | 1 | | 0 | 0 | 6/13 | 0.46 | Weak |
| Butler, 1995 | 0 | 1 | 1 | | 0 | | 0 | | 0 | | 1 | | 0 | | 1 | | 0 | | | 1 | | 1 | | 0 | 1 | 7/16 | 0.44 | Weak |
| Wright, 1996 | 0 | 1 | 1 | | 1 | | 0 | | 0 | | 1 | | 0 | | 0 | | 1 | | | 1 | | 1 | | 0 | 0 | 7/16 | 0.44 | Weak |
| McCloskey, 2008 | 0 | 0 | 0 | | 1 | | N/A | | N/A | | 0 | | 0 | | 1 | | 1 | | | 1 | | 1 | | N/A | 0 | 5/12 | 0.42 | Weak |
| Connor, 2006 | 0 | 1 | 0 | | 1 | | 0 | | 0 | | 0 | | 0 | | 0 | | 1 | | | 1 | | 1 | | 0 | 1 | 6/16 | 0.38 | Weak |
| **^1^Total Points:** No. of points possible= (16 – N/A)  **Key**:  < 0.50= weak; 0.51-0.65= moderate-weak; 0.66-0.79= moderate-strong; > 0.80= strong DV=Dependent Variable; CI=Confidence Interval | | | | | | | | | | | | | | | | | | | | | | | | | | | | |
