# Supplementary material for: Individual determinants of research utilization by nurses: a systematic review update
Source: Implement Sci. 2011 Jan 5;6:1. doi: 10.1186/1748-5908-6-1 (PMC3024963; doi:10.1186/1748-5908-6-1)
Supplement: Additional file 3 — Quality assessment for the included quasi-experimental articles. A description of the findings from the quality assessment of included articles describing studies that used a quasi-experimental study design. [file 1748-5908-6-1-S3.DOC]

**Additional File 3. Quality assessment for the included quasi-experimental articles (n =** 2)

| **First Author, Year** | **Selection Bias** | **Allocation Bias** | **Confounders** | **Blinding** | **Data Collection Methods** | **Withdrawals and Drop-Outs** | **Total Points^1^** | **Score** | **Quality** |
| --- | --- | --- | --- | --- | --- | --- | --- | --- | --- |
| Tranmer, 2002 | Moderate | Moderate | Strong | Not applicable | Strong | Weak | 11/5 | 2.2 | Moderate-Weak |
| Tsai, 2003 | Weak | Moderate | Weak | Not applicable | Moderate | Strong | 9/5 | 1.8 | Weak |
| **^1^ Total Points:** 6 – the number of points not applicable for the article  **Key:**  Weak (1.0-2.0), moderate-weak (2.10-2.34), moderate-strong (2.35-2.66), or strong (2.67-3.00) | | | | | | | | | |
