# Supplementary material for: Individual determinants of research utilization by nurses: a systematic review update
Source: Implement Sci. 2011 Jan 5;6:1. doi: 10.1186/1748-5908-6-1 (PMC3024963; doi:10.1186/1748-5908-6-1)
Supplement: Additional file 4 — Characteristics of the included studies. A detailed summary of the characteristics of all articles included in the review. [file 1748-5908-6-1-S4.DOC]

**Additional File 4. Characteristics of the included studies (n** = 45)

| **First Author, Journal, Year** | **Design** | **Sample/Subjects** | **Setting/Location** | **Framework** | **Research Utilization Instrument** | | | |
| --- | --- | --- | --- | --- | --- | --- | --- | --- |
|  |  |  |  |  | **Name** | **Description/Scoring** | **Reliability** | **Validity** |
| Barta, Journal of Professional Nursing, 1995 | Cross-sectional | **Sample size:**  n **=** 213  **Subjects:** Pediatric nurse educators | **Setting:** 409 baccalaureate degree programs in nursing  **Country:** United States | Roger’s (1983) Theory of Diffusion of Innovations | Modified NPQ^1^-Education (NPQ-E) | Multiple items. Scored dichotomous yes/no for all questions and sometimes/always for the question on use  Total Innovation Adoption Behavior (TIAB) score calculated to categorize participants’ stage of adoption | α = 0.74 | **Content:** expert panel of three paediatric nurses active in paediatric pain assessment and management by reviewing the research base for each practice. |
| Berggren, Journal of Advanced Nursing, 1996 | Cross-sectional | **Sample size:** n **=** 108 (returned)  n = 84 (completed)  **Subjects:** Swedish Midwives | **Setting:** Members of a county division of the Swedish Midwives Association  **Country:** Sweden | Roger’s (1983) Theory of Diffusion of Innovations | Modified NPQ^1^  The Midwifery Practice Questionnaire (MPQ) | Multiple items. Scored dichotomous yes/no for all questions and sometimes/always for the question on use  Total Innovation Adoption Behavior (TIAB) score calculated to categorize participants’ stage of adoption | α (Pilot, n = 25) = 0.79  α (current study) = 0.68  α (subscales) = 0.59 to 0.76 | **Content:** midwifery practices taken from doctoral dissertations and articles published in the Journal of the Swedish Midwives’ Association |
| Bonner, Journal of Nursing Management, 2008 | Cross-sectional | **Sample size:** n = 347  **Subjects:** Registered and enrolled nurses | **Setting:** Cairns District health Services (CDHS)  Includes a regional hospital, two rural hospitals, two health centres, and community health facilities  **Country:**  Australia | Not specified | Edmonton Research Orientation Survey (EROS^2^)  Using Research/Evidence-Based Practice subscale | Ten items scored on a 5-point Likert scale: strongly disagree (1) to strongly agree (5).  Overall score = mean of 10 items | α (current study, EROS) = 0.95 | Construct (current study)-factor analysis with three retained components  (45.1% explained variance in total):  1) Attitude (18.0%)  2) Use of Research (15.6%)  3) Knowledge of Research (11.4%) |
| Bostrom, Implementation Science, 2008 | Cross-sectional | **Sample size:** n = 140 (descriptive) n = 134 for correlations (data from six respondents could not be used due to >50% missing items in the RU Index)  **Subjects:** Registered nurse working in the care of older people | **Setting:** Multiple sites in eight municipalities for Elder care including nursing homes, rehabilitation units, and group dwellings  **Country:**  Sweden | Not specified | Research Utilization Questionnaire (RUQ^3^)  Using Research Subscale | Ten items scored on a 5-point Likert scale: strongly disagree (1) to strongly agree (5).  Overall score = mean of 10 items | α (current study, RU index) = 0.84 | Not reported |
| Brett, Journal of Continuing Education in Nursing, 1987 | Cross-sectional | **Sample size:** n = 216  **Subjects:** Registered Nurses | **Setting:** 19 Acute care hospitals: medical, surgical, or intensive care units  **Country:**  United States | Roger’s (1983) Theory of Diffusion of Innovations | The Nursing Practice Questionnaire (NPQ^1^) | Scored dichotomous yes/no for all questions and sometimes/always for the question on use  Total Innovation Adoption Behavior (TIAB) score calculated to categorize participants’ stage of adoption | α (pilot) = 0.82  Test-retest (pilot, one-week interval)  r = 0.83    α (current, NPQ) = 0.95  α (current, 14 subscales) = 0.68 to.95 | **Content:** Assumed as the innovations were derived from published research reports using specific criteria developed by Haller *et al.* 1979 |
| Brown, Journal of Continuing Education in Nursing,1997 | Cross-sectional | **Sample size:** n = 753  **Subjects:** Nurses | **Setting:** 29 health care facility locations  **Country:**  United States | Not specified | Nursing Research Utilization Survey (developed for this study)  Single item | Number of times participated in activity | Not reported | Not reported |
| Butler, The Canadian Journal of Nursing Research, 1995 | Cross-sectional | **Sample size:** n = 348  **Subjects:** Staff nurses, head nurses, clinical nurse specialists, nurse educators, hospice nurses, expanded-role nurses, and enterostomal therapy nurses | **Setting:**  One large tertiary hospital (Victoria General Hospital, Nova Scotia)  **Country:**  Canada | Not specified | Research Survey  (developed for this study)    Single item | Scored dichotomous yes/no | Not reported | Not reported |
| Champion, Journal of Advanced Nursing, 1989 | Cross-sectional | **Sample size:** n = 59  **Subjects:** Registered nurses (medical, surgical, labour/delivery, postpartum, nursery, ICU, CCU) | **Setting:**  One community hospital  **Country:**  United States | Not specified | Research Utilization Questionnaire (RUQ^3^)  Using Research Subscale | Ten items scored on a 5-point Likert scale: strongly disagree(1) to strongly agree(5) | α (sub-scales) = 0.84 to 0.94  α (overall) = 0.92  α (use subscale) = 0.92 | **Content**- expert panel |
| Connor, 2006 (dissertation) | Cross-sectional | **Sample size:** n = 143  **Subjects:** Registered nurses (n = 39), licensed practice nurses (n = 31), personal care workers (n = 73) | **Setting:**  Number of sites: 12 (five urban, seven rural) nursing home facilities  **Country:**  Canada | Not specified | Research Utilization Survey (adapted from Estabrooks 1999)  Single items for four kinds of research utilization: instrumental, conceptual, persuasive, overall | Scored on a 7-point response scale:  1 = never  2 = on one or two shifts  3 unlabelled  4 unlabelled  5 = on about half of the shifts  6 unlabelled  7 = nearly every shift  8 = do not know | Not reported | **Content**-pilot study with six individuals from each of the three groups |
| Coyle, Nursing Research, 1990 | Cross-sectional | **Sample size:** n = 113  **Subjects:** Registered nurses, registered practical nurses | **Setting:**  Ten acute care hospital  **Country:**  United States | Roger’s (1983) Theory of Diffusion of Innovations | The Nursing Practice Questionnaire (NPQ^1^) | Multiple items. Dichotomous yes/no for all questions and  sometimes/always  for the question on use | α (NPQ) = 0.91  α (14 subscales) = 0.79-.90 | **Content**—Nursing practices from published literature (replication of Brett 1987 study) |
| Cummings, Nursing Research, 2007 | Secondary analysis of cross-sectional data | **Sample sizes after listwise deletion**  1998 dataset (n = 3,701)  **Used in analysis** (n = 1,200; *i.e.*, 300 cases per context group)  **Subjects:** Registered nurses | **Setting:** All RNs in Alberta Canada  **Country:**  Canada | PARiHS | Questionnaire (same data as Estabrooks 1999)  Single item | Scored on a 7-point response scale:  1 = never  2 = on one or two shifts  3 unlabelled  4 unlabelled  5 = on about half of the shifts  6 unlabelled  7 = nearly every shift  8 = do not know | Not reported | **Content**—Derived measure developed based on predictors of research utilization found in the literature.  **Content—**Development of the theoretical model was guided by the PARIHS framework, the literature, previous research, and administrative experience |
| Erler, Air Medical Journal, 2000 | Cross-sectional | **Sample size:** n = 497  **Subjects:** Nurses  (Air and Surface Transport) | **Setting:**  Members of the Air and Surface Transport Nurses Association (ASTNA)  **Country:**  United States | Not specified | Questionnaire (developed for this study)  Single item | Dichotomous yes/no | Not reported | Not reported |
| Estabrooks, Research in Nursing and Health, 2007 | Cross-sectional | **Sample size: Canadian-**n = 600  **US Army-**n = 290  **Subjects:** Nurses | **Setting:**  Canada: health care settings in Alberta (mainly hospitals)  United States: three US Army hospitals in North east  **Country:**  Canada and United States | Not specified | Questionnaire  (adapted from Estabrooks, 1999)  Single items for two kinds of research utilization: instrumental, overall | Scored on a 7-point response scale:  1 = never  2 = on one or two shifts  3 unlabelled  4 unlabelled  5 = on about half of the shifts  6 unlabelled  7 = nearly every shift  8 = do not know | Not reported | No new data presented  Refers to Estabrooks 1999 |
| Estabrooks, Western Journal of Nursing Research,1999 | Cross-sectional | **Sample size:** n = 600  **Subjects:** Registered nurses-direct patient care | **Setting:**  Members of the Alberta association Registered Nurses  **Country:**  Canada | Roger’s (1983) Theory of Diffusion of Innovations | Questionnaire  (developed for this study)  Single item | Scored on a 7-point response scale:  1 = never  2 = on one or two shifts  3 unlabelled  4 unlabelled  5 = on about half of the shifts  6 unlabelled  7 = nearly every shift  8 = do not know | Not reported | **Content:**  —reviewed by two researchers with expertise in the field.  —Careful attention paid to theoretical conceptualizations of research utilization in the literature, questioning approaches of previous investigators, theoretical needs of the study, and the investigator’s clinical experience.  —Pilot testing on a convenience sample (n = 23) of post-basic baccalaureate nursing students and master’s nursing students. The labeling convention was chosen as pilot testing suggested that concrete labels were required to make explicit that the numerical scale was a relative scale |
| Forbes, Journal of Nursing Measurement, 1997 | Cross-sectional | **Sample size:** n = 1,117  **Subjects:** Staff RNs (medical/surgical, critical care, operating/recovery room, obstetrics/gynecology, and others) | **Setting:** Four acute care hospitals in the Midwest  **Country:** United States | Not specified | Control Over Nursing Practice Instrument | Scored on a 4-point scale:  0  1  2-4  5 or more times  Does not apply | α (RU subscale) = 0.78 | **Dimensionality:**  High factor loadings for research use subscale: (0.66 to 0.82) |
| Hatcher, Canadian Journal of Nursing Administration, 1997 | Cross-sectional | **Sample size:** n = 174  NAC members (n = 37)  Staff (n = 137)  **Subjects:** Registered nurses, registered practical nurses | **Setting:**  acute care hospital  **Country:**  Canada | Not specified | Research Utilization Questionnaire (RUQ^3^)  Using Research Subscale | Ten items scored on a 5-point Likert scale: strongly disagree (1) to strongly agree(5) | Not reported | Not reported |
| Humphris, Practical Diabetes International, 1999 | Cross-sectional | **Sample size:**  DNS n = 299  NNS n = 133  **Subjects:** Registered Nurses-Diabetic Nurse Specialists (DNS) and Non-Nurse specialists (NNS) | **Setting:**  acute care trusts  **Country:**  United Kingdom | Not specified | Questionnaire  (developed for this study)  single item | Dichotomous yes/no | Not reported | Not reported |
| Kenny, Canadian Journal of Nursing Leadership, 2005 | Cross-sectional | **Sample size:** n = 290  Military(160)  Civilian (130)  **Subjects:** Registered nurses  (Military and Civilian) | **Setting:**  Three hospitals in the North Atlantic Regional Medical Command  **Country:**  United States | Not specified | Research Utilization Survey (Adapted from Estabrooks, 1999)  Single items for four kinds of research utilization: instrumental, conceptual, persuasive, overall | Scored on a 7-point response scale:  1 = never  2 = on one or two shifts  3 unlabelled  4 unlabelled  5 = on about half of the shifts  6 unlabelled  7 = nearly every shift  8 = do not know | Not reported | Not reported |
| Lacey, Journal of Advanced Nursing, 1994 | Cross-sectional pilot | **Sample size:** n = 20  **Subjects:** Registered nurses in the United Kingdom | **Setting:**  two hospitals; adult acute areas.  Hospital A: district general hospital in an industrial town  Hospital B: a high-profile teaching hospital in a major city  **Country:**  United Kingdom | Not specified | Research Utilization Questionnaire (RUQ^3^)  Using Research Subscale | Ten items scored on a 5-point Likert scale: strongly disagree(1) to strongly agree(5) | Not reported | Not reported |
| Logsdon, Kentucky Nurse, 1998 | Cross-sectional | **Sample size:** n = 196  **Subjects:** Nurses registered with the Kentucky Board of Nursing | **Setting:**  Kentucky  **Country:**  United States | Not specified | Registered Nurses' Views on Research (developed for this study) | Not reported | Not reported | **Content:** based on the literature and the investigators experience with research use in the clinical setting |
| McCleary, Nurse Education Today, 2003 | Cross-sectional | **Sample size:** n = 175  **Subjects:** Registered nurses | **Setting:**  One paediatric acute care hospital  **Country:**  Canada | Not specified | Edmonton Research Orientation Survey (EROS^2^)  Using Research/Evidence-Based Practice subscale | Ten items scored on a 5-point Likert scale: strongly disagree (1) to strongly agree (5). | Not reported | **Construct**: State that construct validity of the subscales were good |
| McCleary, Journal of Nursing Measurement, 2002 | Cross-sectional | **Sample size:** n = 185  **Subjects:** Registered Nurses | **Setting:**  One Paediatric teaching hospital  **Country:**  Canada | Not specified | Edmonton Research Orientation Survey (EROS^2^)  Using Research/Evidence-Based Practice subscale | Ten items scored on a 5-point Likert scale: strongly disagree (1) to strongly agree (5). | α (EROS) = 0.94  α (EBP subscale) = 0.87 | Not reported |
| McCloskey, 2005 (dissertation)  McCloskey, Journal of Nursing Scholarship, 2008 | Cross-sectional | **Sample size:** n = 270  **Subjects:** All registered nurses >18 years of age working within the Iowa hospital system | **Setting:**  Five Iowa hospitals  **Country:**  United States | Not specified | Research Utilization Questionnaire (RUQ^3^)  Using Research Subscale | Ten items scored on a 5-point Likert scale: strongly disagree(1) to strongly agree(5) | α (RU subscale) = 0.93 | No new data reported  Refers to Champion and Leach 1989 |
| Michel, Journal of Professional Nursing, 1995 | Cross-sectional | **Sample size:** n = 167 (returned)  n = 157 (completed)  **Subjects:** Nurses | **Setting:**  Members of STTI Honor Society associated with a university in a metropolitan setting  **Country:**  United States | Roger’s (1983) Theory of Diffusion of Innovations | Modified Nursing Practice Questionnaire (NPQ^1^) | Multiple items. Dichotomous yes/no for all questions and sometimes/always for the question on use | α (NPQ) = 0.85    α(subscales) = 0.73 to 0.84 | **Content**- assumed as research findings derived from published nursing literature using specific criteria |
| Milner, International Journal of Nursing Studies, 2005 | Cross-sectional | **Sample size:** n **=** 389  **Subjects:** Staff nurses, educators and managers | **Setting:**  Nurses registered with the Alberta Association of registered nurses in Alberta, Canada  **Country:**  Canada | Not specified | Research Utilization Survey (Adapted from Estabrooks, 1999)  Single items for four kinds of research utilization: instrumental, conceptual, persuasive, overall | Modified response scale used. Scored on a 5-point response scale: never (1) to nearly every day (5) | Not reported | Not reported |
| Nash, 2005 (Dissertation) | Cross-sectional | **Sample size:** n = 82  **Subjects:** Registered Nurses — Nurses registered with the Idaho State Board of Nursing | **Setting:**  State of Idaho  **Country:**  United States | Not specified | Utilities #2 questionnaire | Multiple items. Scored on a 4-point Likert-type scale:  Strongly disagree  Disagree  Agree  Strongly agree | α (utilization subscale) = 0.917 | Not reported |
| Ofi, International Journal of Nursing Practice, 2008 | Cross-sectional | **Sample size: Whole sample**  n = 500    **By hospital**  (n = 199, 162, and 139)  **Subjects:** Nurses in Nigeria | **Setting:**  three Tertiary hospitals  **Country**:  Nigeria | Not specified | Research Conduct and Research Utilization by Nurses Questionnaire (developed for this study)    Single item | Scored on a 5-point scale:  Never  Seldom  Sometimes  Frequently  All the time | Not reported | **Content**—experts in the field |
| Parahoo, Journal of Advanced Nursing, 1999 | Cross-sectional | **Sample size:**  n = 1,368  **Subjects:** Hospital nurses in Northern Ireland | **Setting:**  23 Hospitals in 14 Trusts  **Country:**  United Kingdom (Ireland) | Not specified | Questionnaire (developed for this study)  Single item | Scored on a 5-point scale:  Never  Seldom  Sometimes  Frequently  All the time | States that it was ‘piloted for reliability with a group of 20 nurses’ | **Content**-panel of three experts  **Content**-questionnaire developed after a review of the literature on research utilization and research activities |
| Parahoo, Journal of Nursing Management, 2001 | Cross-Sectional | **Sample size:** n = 479  n = 1,368 (total sample)  **Subjects:** Medical/surgical nurses (subset of results from Parahoo 1998) | **Setting:** Ten hospitals  **Country:**  United Kingdom (Ireland) | Not specified | Questionnaire (developed for this study)  Single item | Scored on a 5-point scale:  Never  Seldom  Sometimes  Frequently  All the time | not reported | **Content**-panel of three experts  **Content**-questionnaire developed after a review of the literature on research utilization and research activities  Reports a pilot with 20 nurses |
| Prin, Studies in Health Technology and Informatics, 1997 | Cross-sectional | **Sample size:** n = 121  **Subjects:** Female clinical nurses | **Setting:** medical-surgical units in one large, university medical center  **Country:**  United States | Not specified | Modified Research Utilization Questionnaire (RUQ^3^)  Using Research Subscale | Ten items scored on a 5-point Likert scale: strongly disagree (1) to strongly agree (5) | α = 0.942  Pilot testing indicated one item contributed to low reliability. This item was removed from the scale | **Content**—by three nursing informatics experts |
| Profetto-McGrath, Western Journal of Nursing Research, 2003 | Cross-sectional | **Sample size:** n = 141 (valid responses from a total of 143 returned)  **Subjects:** Registered Nurses | **Setting:**  Seven hospitals; Adult surgical (n = 2) and pediatric units (n = 5)  **Country:** Canada | Roger's (1983) Theory of Diffusion of Innovations | Research Utilization Survey  (shortened version of Estabrooks, 1999)  Single items for four kinds of research utilization: instrumental, conceptual, persuasive, overall | Scored on a 7-point response scale:  1 = never  2 = on one or two shifts  3 unlabelled  4 unlabelled  5 = on about half of the shifts  6 unlabelled  7 = nearly every shift  8 = do not know | Not reported | No new data presented  Refers to Estabrooks 1999 |
| Profetto-McGrath, Nurse Education in Practice, 2009 | Cross-Sectional | **Sample size:** n = 287  **Subjects:** Nurse educators | **Setting:**  Members of a provincial association of registered nurses in western Canada  **Country:**  Canada | Not specified | Research Utilization Survey  (shortened version of Estabrooks, 1999)  Single items for four kinds of research utilization: instrumental, conceptual, persuasive, overall | Modified response scale used. Scored on a 5-point response scale: never (1) to nearly every day (5) | Not reported | No new data presented  Refers to Estabrooks 2008 |
| Rodgers, Nurse Education Today, 2000  (a study..) | Cross-sectional | **Sample size:** n = 680  **Subjects:** Registered nurses-general medical and surgical wards | **Setting:**  25 Hospitals in the Scottish Health Service  **Country:**  United Kingdom (Scotland) | Roger's (1983) Theory of Diffusion of Innovations | Modified Nursing Practice Questionnaire (NPQ^1^) | Multiple items. Dichotomous yes/no for all questions and sometimes/always for the question on use | α (mean research utilization score over all of the 14 practices) = 0.631 | **Content**-panel of nurse researchers and educators.  **Construct-**authors report that, as the 14 practices and influencing factors identified in the earlier exploratory study, the survey was felt to have construct validity  **Content-**validity of self-reporting levels of research utilization confirmed in pilot with 20 nurses |
| Rutledge, Oncology Nursing Forum, 1996 | Cross-sectional | **Sample Size:** n = 1,100 ONS members (n = 769)  Networking (n = 331)  **Subjects:** Staff nurses (oncology) | **Setting:** Oncology settings (hospitals, comprehensive cancer center, outpatient care clinic, hospice, home care, private/group practice, physician’s office)  **Country:**  United States | Roger's (1983) Theory of Diffusion of Innovations | Modified Nursing Practice Questionnaire^1^—-  The Oncology Nursing  Practice Questionnaire (ONPQ) | Multiple items. Dichotomous yes/no for most questions and sometimes/always for the question on use | α (ONPQ overall) = 0.75 | Not reported |
| Squires, Implementation Science, 2007 | Cross-sectional | **Sample size:** n = 248 **Subjects:** Registered nurses — medical, surgical, and/or critical care nurses | **Setting:** adult acute care hospitals  **Country:**  Canada | Roger's (1983) Theory of Diffusion of Innovations | Modified Nursing Practice Questionnaire^1^ | Multiple items. Dichotomous yes/no for most questions and sometimes/always for the question on use  Total Innovation Adoption Behavior (TIAB) score calculated to categorize participants’ stage of adoption | α (modified NPQ) = 0.82 | **Content —** assumed as the research-based practices selected were identified from  existing research literature using specific criteria |
| Stiefel, 1996 (dissertation) | Cross-sectional | **Sample size:** n = 100 **Subjects:** Clinical nurses from adult medical, oncology, surgical, and critical care | **Setting:** 20 nursing units in two university-affiliated teaching hospitals  **Country:**  United States | Item selection guided by Rogers (1983), CURN (Horseley *et al.* 1983), and the Iowa Model of Research Use in Practice (Titler *et al.* 1994) | Nursing Research  Utilization Survey  (NRUS) (developed for this study) | Multiple items scored on a 5-point Likert scale:  Never  Seldom  Sometimes  Frequently  Always  Scoring range: 18 to 90 | Reliability  Test-retest of a convenience sample of 257 nurses from three hospitals to develop the NRUS.  Involved a test-retest of this convenience sample (see below)  Test segment:  N = 211 RNs (RR: 82.1%)  Answered all 20 items of the NRUS: n = 202,  α = 0.941  Re-test segment:  n = 188 RNs (RR: 89.1%)  Answered all 20 items of the NRUS: n = 176  α = 0.951  Reliability correlation  of the two scores  r = 0.876 | **Content (pilot)**-by four NRU experts (two members of the CURN project, one developer of the Iowa model, and one who works actively with nurses on NRU projects)  **Content (current study)**—Clinical nurse researcher at the Midwest site  **Construct:**-  factor analysis  (n = 202 RNs)  2 items were deleted  from the original 20 item survey based on factor analysis |
| Tranmer, Canadian Journal of Nursing Leadership, 2002 | Quasi-experimental | **Sample size =** All Nursing  Pretest  (n = 92)  Post-test (n = 88) (High, Low, control)  Pretest: (n = 37, n = 21, n = 34)  Post-test: (n = 29, n = 39, n = 24)  Working Group (High, Low)  Pre-test (n = 18, n = 6)  Post-test (n = 17, n = 4)  **Subjects:** Registered Nurses — high, low, and controlled exposure to research | **Setting:** One acute care hospital  **Country:**  Canada | Not specified | Research Utilization Questionnaire (RUQ^3^)  Using Research Subscale | Ten items scored on a 5-point Likert scale: strongly disagree(1) to strongly agree(5) | α (sub-scales) = 0.85 to 0.94  α (use sub-scale) = 0.93 | Not reported |
| Tsai, International Journal of Nursing Studies, 2000 | Cross-sectional | **Sample size =** Staff nurses (n = 271)  Managers (n = 111)  **Subjects:** Staff Nurses and Nurse Managers | **Setting:**  largest medical center in Taipei  **Country:**  Republic of China (*i.e.*, Taiwan) | Not specified | Research Utilization questionnaire  (adapted from Funk *et al.* 1991 and Pettengill *et al.* 1994)  Single item | Dichotomous- yes/no | Not reported | **Content**-expert panel of eight nurses prepared at masters and doctoral levels  **Content**- pilot test to ensure the tool content was associated with other data reported in the literature and was sensitive to the symbolic meanings relevant in Taiwan’s nursing community |
| Tsai, International Journal of Nursing Studies, 2003 | Quasi-experimental | **Sample size:** Control group (n = 42)  Experimental group (n = 47)  **Subjects:** Nurses with at least one year of working experience | **Setting:**  A medical center  **Country:**  Taiwan | Not specified | Research Utilization questionnaire (adapted by Tsai 2000 from Funk *et al.* 1991 and Pettengill *et al.* 1994)  Single item | Dichotomous- yes/no | Not reported | **Content**-tool checked and confirmed by five clinical nurses |
| Varcoe, Canadian Journal of Nursing Research, 1995 | Cross-sectional | **Sample size:** n = 183  **Subjects:** Registered Nurses | **Setting:** medical surgical and critical care areas of acute care hospitals  **Country:**  Canada | Roger’s (1983) Theory of Diffusion of Innovations | The Research Use in Nursing Practice Instrument (Alcock 1990; modified by Clarke 1991)  and  Nursing Practice Questionnaire (NPQ^1^) | General use:  Ten items scored on a 4-point scale:  Not at all  2 = Sometimes  3 = Frequently  4 = Always  Possible score range 10 to 40  NPQ  Use of 10 specific findings: 3-point scale:  1 = Never  2 = Sometimes  3 = Always  ‘not applicable’ | General research use  α = 0.87  NPQ  α = 0.87 | **Content** (total instrument)-pilot testing and peer review |
| Wallin, Journal of Advanced Nursing, 2003 | Cross-sectional | **Sample size:** n = 119  QI+(n = 46)  QI- (n = 72)  **Subjects:** Registered nurses who participated in a QI training course | **Setting:** Various clinical areas (>75) -acute care, psychiatry, primary care, and nursing homes    **Country:**  Sweden | Not specified | Research Utilization Questionnaire (RUQ)  Using Research Subscale  Additional item  (Pettengill 1994) | Ten items scored on a 5-point Likert scale: strongly agree (1)-strongly disagree (5)    Dichotomous yes/no on additional item | Not reported (for current study) | Not reported |
| Wallin, Nursing Research, 2006 | secondary analysis of cross-sectional data | **Sample sizes:** S**ample sizes after listwise deletion**  **1996**  (n = 504)  **1998** (n = 5946)  **Subjects:** Registered Nurses | **Setting:** Any setting that a registered nurse may work (random sample of all registered nurses in one Canadian province)  **Country:**  Canada | PARiHS | Questionnaire (same data as Estabrooks 1999)  Single item for overall research utilization | Scored on a 7-point response scale:  1 = never  2 = on one or two shifts  3 unlabelled  4 unlabelled  5 = on about half of the shifts  6 unlabelled  7 = nearly every shift  8 = do not know | Not reported | **Content**—Derived measure developed based on predictors of research utilization found in the literature. |
| Wells, Clinical Nurse Specialist: The Journal for Advanced Nursing Practice, 1994 | Cross-sectional | **Sample sizes: Whole sample** (n = 279)  **Staff Nurses**  (n = 156)  **Managers** (n = 37)  **Advanced Practice Nurses (CNS and NPs)**  (n = 86)  **Subjects:** Nurses working in a large academic medical centre | **Setting:**  One large academic medical centre  **Country:**  United States | Not specified | Questionnaire (developed for this study)  Single item | Dichotomous yes/no | Not reported | Not reported |
| Wright, Australian Journal of Advanced Nursing, 1996 | Cross-sectional | **Sample size:** n = 410  **Subjects:** Registered nurses-general and psychiatric | **Setting:** General nurses from three teaching hospitals in Sydney; psychiatric nurses from a large Sydney hospital, a medium sized private psychiatric hospital, or from community mental health centres in Sydney  **Country:**  Australia | Not specified | Questionnaire (designed for this study)  Single item | Not reported — appears to be dichotomous yes/no | Not reported | **Content**-consultation with three clinical nurse consultants |

^1^NPQ = Nurses Practice Questionnaire. NPQ consists of a series of questions following each of several research-based innovations. Scores on individual items are combined to obtain an overall Innovation Adoption Score, based on Rogers (1983) Diffusion of Innovations Theory

^2^EROS = Edmonton Research Orientation Survey. EROS consists of four subscales of which ‘Using Research/Evidence-Based Practice’ is one subscale. This subscale is composed of 10 items measuring general research use.

^3^RUQ = research Utilization Questionnaire. The RUQ consists of 42 self-descriptive statements comprising four subscales of which research use is one. The research use subscale contains 10 items, which measure the degree to which an individual feels they incorporate research findings into their daily practice.
